# Are we ready to track climate‐driven shifts in marine species across international boundaries? ‐ A global survey of scientific bottom trawl data

**DOI:** 10.1111/gcb.15404

**Published:** 2020-11-10

**Authors:** Aurore A. Maureaud, Romain Frelat, Laurène Pécuchet, Nancy Shackell, Bastien Mérigot, Malin L. Pinsky, Kofi Amador, Sean C. Anderson, Alexander Arkhipkin, Arnaud Auber, Iça Barri, Richard J. Bell, Jonathan Belmaker, Esther Beukhof, Mohamed L. Camara, Renato Guevara‐Carrasco, Junghwa Choi, Helle T. Christensen, Jason Conner, Luis A. Cubillos, Hamet D. Diadhiou, Dori Edelist, Margrete Emblemsvåg, Billy Ernst, Tracey P. Fairweather, Heino O. Fock, Kevin D. Friedland, Camilo B. Garcia, Didier Gascuel, Henrik Gislason, Menachem Goren, Jérôme Guitton, Didier Jouffre, Tarek Hattab, Manuel Hidalgo, Johannes N. Kathena, Ian Knuckey, Saïkou O. Kidé, Mariano Koen‐Alonso, Matt Koopman, Vladimir Kulik, Jacqueline Palacios León, Ya’arit Levitt‐Barmats, Martin Lindegren, Marcos Llope, Félix Massiot‐Granier, Hicham Masski, Matthew McLean, Beyah Meissa, Laurène Mérillet, Vesselina Mihneva, Francis K. E. Nunoo, Richard O'Driscoll, Cecilia A. O'Leary, Elitsa Petrova, Jorge E. Ramos, Wahid Refes, Esther Román‐Marcote, Helle Siegstad, Ignacio Sobrino, Jón Sólmundsson, Oren Sonin, Ingrid Spies, Petur Steingrund, Fabrice Stephenson, Nir Stern, Feriha Tserkova, Georges Tserpes, Evangelos Tzanatos, Itai van Rijn, Paul A. M. van Zwieten, Paraskevas Vasilakopoulos, Daniela V. Yepsen, Philippe Ziegler, James T. Thorson

**Affiliations:** ^1^ Centre for Ocean Life National Institute of Aquatic Resources (DTU Aqua) Technical University of Denmark Kgs. Lyngby Denmark; ^2^ Section for Ecosystem based Marine Management National Institute of Aquatic Resources (DTU Aqua) Technical University of Denmark Kgs. Lyngby Denmark; ^3^ Aquaculture and Fisheries Group Wageningen University & Research Wageningen The Netherlands; ^4^ Norwegian College of Fishery Science UiT The Arctic University of Norway Tromsø Norway; ^5^ Fisheries and Oceans Canada Bedford Institute of Oceanography Dartmouth NS Canada; ^6^ MARBEC University of Montpellier CNRS Sète France; ^7^ Department of Ecology, Evolution, and Natural Resources Rutgers, The State University of New Jersey New Brunswick NJ USA; ^8^ Fisheries Scientific Survey Division Fisheries Commission Tema Ghana; ^9^ Fisheries and Oceans Canada Pacific Biological Station Nanaimo BC Canada; ^10^ Falkland Islands Fisheries Department Directorate of Natural Resources Stanley Falkland Islands; ^11^ Halieutique Manche Mer du Nord unit French Research Institute for the Exploitation of the Sea (IFREMER) Boulogne‐sur‐Mer France; ^12^ Centro de Investigaçao Pesqueira Aplicada (CIPA) Bissau Guinea‐Bissau; ^13^ The Nature Conservancy Narragansett RI USA; ^14^ School of Zoology and The Steinhardt Museum of Natural History Tel Aviv Israel; ^15^ Wageningen Marine Research IJmuiden The Netherlands; ^16^ Halieute National Center of Fisheries Sciences of Boussoura Conakry Republic of Guinea; ^17^ General Directorate of Demersal and Coastal Resources Research Instituto del Mar Perú (IMARPE) Callao Perú; ^18^ Fisheries Resources Research Center National Institute of Fisheries Science Tongyeong‐si Korea; ^19^ Greenland Institute of Natural Resources Nuuk Greenland; ^20^ Resource Assessment and Conservation Engineering, Alaska Fisheries Science Center, National Marine Fisheries Service NOAA Seattle WA USA; ^21^ COPAS Sur‐Austral Departamento de Oceanografía University of Concepcion Concepción Chile; ^22^ Fisheries and Aquaculture Biologist ISRA CRODT Dakar Senegal; ^23^ Recanati Institute for Maritime Studies and Department of Maritime Civilizations Charney School of Marine Sciences University of Haifa Haifa Israel; ^24^ Møreforsking Ålesund AS Ålesund Norway; ^25^ Millennium Nucleus of Ecology and Sustainable Management of Oceanic Islands (ESMOI) Departamento de Oceanografía Facultad de Ciencias Naturales y Oceanográficas Universidad de Concepción Concepción Chile; ^26^ Department of Forestry, Fisheries and the Environment Cape Town South Africa; ^27^ Thuenen Institute of Sea Fisheries Bremerhaven Germany; ^28^ Narragansett Laboratory National Marine Fisheries Service Narragansett RI USA; ^29^ Departamento de Biologia Universidad Nacional de Colombia Bogotá Colombia; ^30^ ESE, Ecology and Ecosystem Health Institut Agro Rennes France; ^31^ Ecosystem Oceanography Group (GRECO) Instituto Español de Oceanografía Centre Oceanogràfic de les Balears Palma de Mallorca Spain; ^32^ National Marine Information and Research Centre Ministry of Fisheries and Marine Resources (MFMR) Swakopmund Namibia; ^33^ Fishwell Consulting Pty Ltd Queenscliff Vic. Australia; ^34^ Institut Mauritanien de Recherches Océanographiques et des Pêches Nouadhibou Mauritania; ^35^ Northwest Atlantic Fisheries Centre Fisheries and Oceans Canada St. John's NL Canada; ^36^ Pacific Branch (TINRO) of Russian Federal Research Institute Of Fisheries and Oceanography (VNIRO) Vladivostok Russia; ^37^ Instituto Español de Oceanografía Cádiz Andalucía Spain; ^38^ Département Adaptations du vivant UMR BOREA Museum National d’Histoire Naturelle Paris France; ^39^ Institut National de Recherche Halieutique Casablanca Morocco; ^40^ Department of Biology Dalhousie University Halifax NS Canada; ^41^ National Museum of Natural History Paris France; ^42^ Ifremer Lorient France; ^43^ Institute of Fish Resources Varna Bulgaria; ^44^ Department of Marine and Fisheries Sciences University of Ghana Accra Ghana; ^45^ National Institute of Water and Atmospheric Research Limited Wellington New Zealand; ^46^ Resource Assessment and Conservation Engineering Division, Alaska Fisheries Science Center NOAA Seattle WA USA; ^47^ Institute for Marine and Antarctic Studies University of Tasmania Hobart Tas. Australia; ^48^ National Higher School of Marine Sciences and Coastal Management Dély Ibrahim Algeria; ^49^ Instituto Español de Oceanografía Centro Oceanográfico de Vigo Vigo Spain; ^50^ Marine and Freshwater Research Institute Reykjavik Iceland; ^51^ Israeli Fisheries Division, Fisheries and Aquaculture Department Ministry of Agriculture Kiryat Haim Israel; ^52^ Resource Ecology and Fisheries Management, Alaska Fisheries Science Center, National Marine Fisheries Service NOAA Seattle WA USA; ^53^ Faroe Marine Research Institute Tórshavn Faroe Islands; ^54^ Israel Oceanographic and Limnological Research Institute Haifa Israel; ^55^ Hellenic Centre for Marine Research (HCMR) Heraklion Greece; ^56^ Department of Biology University of Patras Patras Greece; ^57^ School of Zoology Tel Aviv University Tel Aviv Israel; ^58^ Joint Research Centre (JRC) European Commission Ispra Italy; ^59^ Programa de Doctorado en Ciencias con Mención en Manejo de Recursos Acuáticos Renovables (MaReA) Facultad de Ciencias Naturales y Oceanográficas Universidad de Concepción Concepción Chile; ^60^ Antarctic Conservation and Management Program Australian Antarctic Division Department of Agriculture, Water, and the Environment Kingston Tas. Australia; ^61^ Habitat and Ecological Processes Research Program Alaska Fisheries Science Center, National Marine Fisheries Service NOAA Seattle WA USA

**Keywords:** bottom trawl survey, climate change, demersal fish, fisheries policy, global data synthesis, open science, species distribution, transboundary conservation

## Abstract

Marine biota are redistributing at a rapid pace in response to climate change and shifting seascapes. While changes in fish populations and community structure threaten the sustainability of fisheries, our capacity to adapt by tracking and projecting marine species remains a challenge due to data discontinuities in biological observations, lack of data availability, and mismatch between data and real species distributions. To assess the extent of this challenge, we review the global status and accessibility of ongoing scientific bottom trawl surveys. In total, we gathered metadata for 283,925 samples from 95 surveys conducted regularly from 2001 to 2019. We identified that 59% of the metadata collected are not publicly available, highlighting that the availability of data is the most important challenge to assess species redistributions under global climate change. Given that the primary purpose of surveys is to provide independent data to inform stock assessment of commercially important populations, we further highlight that single surveys do not cover the full range of the main commercial demersal fish species. An average of 18 surveys is needed to cover at least 50% of species ranges, demonstrating the importance of combining multiple surveys to evaluate species range shifts. We assess the potential for combining surveys to track transboundary species redistributions and show that differences in sampling schemes and inconsistency in sampling can be overcome with spatio‐temporal modeling to follow species density redistributions. In light of our global assessment, we establish a framework for improving the management and conservation of transboundary and migrating marine demersal species. We provide directions to improve data availability and encourage countries to share survey data, to assess species vulnerabilities, and to support management adaptation in a time of climate‐driven ocean changes.

## Graphical abstract

While many species are changing their spatial distributions rapidly with climate change, the global capacity to track these changes and then adapt management and policy has been limited. To build this capacity for demersal fishes, we compiled the largest worldwide collection of bottom trawl survey metadata and demonstrate how surveys can be combined to follow species across national and survey boundaries. Open data and transparent scientific assessment can be a key step towards improved management of redistributing transboundary species and stocks.

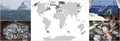

## 1 INTRODUCTION

Marine species worldwide are redistributing in response to climate change and variability (Pinsky et al., 2019; Poloczanska et al., 2013). The movement of individuals from one location to another in response to climate change, either through active migration or passive dispersal of early life stages, results in expansion to unoccupied areas (Dulvy et al., 2008; Perry, 2005). Such redistributions have profound consequences for biodiversity, structures and functions of marine ecosystems (Batt et al., 2017; Friedland et al., 2020; Kortsch et al., 2015; Magurran et al., 2015; McLean et al., 2019; Mérillet et al., 2020). While species on the move have important socio‐economic consequences (Greenan et al., 2019; Pinsky & Fogarty, 2012), our capacity to adapt to these changes by tracking and projecting species range shifts across regional boundaries remains a challenge, not only scientifically, but also economically and politically (Lindegren & Brander, 2018; Pinsky et al., 2018).

The capacity to detect shifts in species ranges depends foremost on the ability to monitor species through, among others, the existence, coverage, and quality of surveys on land and in the oceans (Blowes et al., 2019; Dornelas et al., 2018; Edgar et al., 2017). Among them, scientific bottom trawl surveys were started in the 1900s and collect demersal marine species (living over and on the sea bottom) on continental shelves and slopes in many areas of the world (Garces et al., 2006; Trenkel et al., 2019). The primary purpose of these surveys is to provide fishery‐independent data to inform stock assessment of commercially important demersal populations, and more recently for multidisciplinary ecosystem monitoring. Many of the surveys offer long time series on community composition and provide a unique opportunity to track species range shifts and improve the assessment of biodiversity under global change.

Studies examining climate change impacts on marine communities across large regions have mostly focused on the North Atlantic, Northeast Pacific, and Australian ecosystems (Lenoir et al., 2020; Richardson et al., 2012). This focus is at least partly due to ready availability of ecological surveys in these regions and the lack of knowledge regarding surveys elsewhere. While there is a global movement towards “open science” (OCDE, 2015), particularly by making data publicly available (Cheruvelil & Soranno, 2018; Gallagher et al., 2020; Nosek et al., 2015; Poisot et al., 2019), it has also sparked considerable debate over how to proceed (Moles et al., 2013; Poisot et al., 2013). Therefore, the application of open science principles remains a challenge. From a political and management perspective, there is a need to combine surveys across international boundaries because commercial species spread and redistribute over multiple management areas, requiring transboundary assessments (Baudron et al., 2020; Ramesh et al., 2019). The lack of such assessments may lead to political disputes over shifting fisheries resources (Pinsky et al., 2018; Shackell et al., 2016; Spijkers & Boonstra, 2017). If the data generated by bottom trawl surveys are available and combined properly—they may allow near‐seamless comparisons of species distribution and abundance in time and space. Developing knowledge on marine species responses to climate change is the first step towards developing transboundary and international management plans.

To uncover the difficulties preventing a global assessment of marine species redistributions, we first review the existence and availability of bottom trawl surveys worldwide by collecting survey metadata. We assess the global coverage of productive and trawled seas by bottom trawl surveys. Second, we show the importance of combining surveys to cover commercial species ranges. Third, we demonstrate that modeling can appropriately incorporate multiple surveys to follow species density in time and space. We propose a framework where open science would help to support transboundary management and conservation.

## 2 GLOBAL AVAILABILITY OF TRAWL SURVEY DATA

Scientific bottom trawl surveys have been conducted in many countries in continental waters—sampling demersal fishery resources on continental shelves and slopes. They have formed the backbone of information supporting research on marine fish communities in response to climate change and variability across ecosystems and over large spatial scales (Beukhof et al., 2019; Branch et al., 2010; Gislason et al., 2020; Pecuchet et al., 2017; Pinsky et al., 2013; Shin et al., 2005), as well as meta‐analysis across taxonomic groups and biomes (Antão et al., 2020; Burrows et al., 2019; Lenoir et al., 2020). However, a single bottom trawl survey (henceforth survey) is typically carried out nationally, regionally or within a delimited management zone (but this is not the case for some of the European surveys). As a result, monitoring protocols differ among surveys, and the data are not always publicly available, while the assessment of species range shifts critically depends on the availability and quality of surveys (Costello et al., 2013; Schindler & Hilborn, 2015).

### 2.1 Global data synthesis

To assess the existence and accessibility of fishery‐independent data on demersal species in the global ocean, we collected survey metadata (latitude, longitude, and depth if available) for recent and ongoing surveys that included at least 1 year of sampling since 2015 and use otter trawl gear, the most common gear type. We only retained surveys that were performed for 4 years or more between 2001 and 2019 (complete list in Table S1.1). Finally, we excluded surveys covering near‐shore areas (within 3 miles from the coast) as they primarily target juvenile or coastal fish. Species life cycles are affected by climate change (Petitgas et al., 2013), and surveys providing information on the different life stages of shifting species—such as near‐shore surveys—could constitute a complementary global source of survey data to the one presented here. We recorded all surveys that did not meet our criteria (Table S2.1).

**FIGURE 1:**
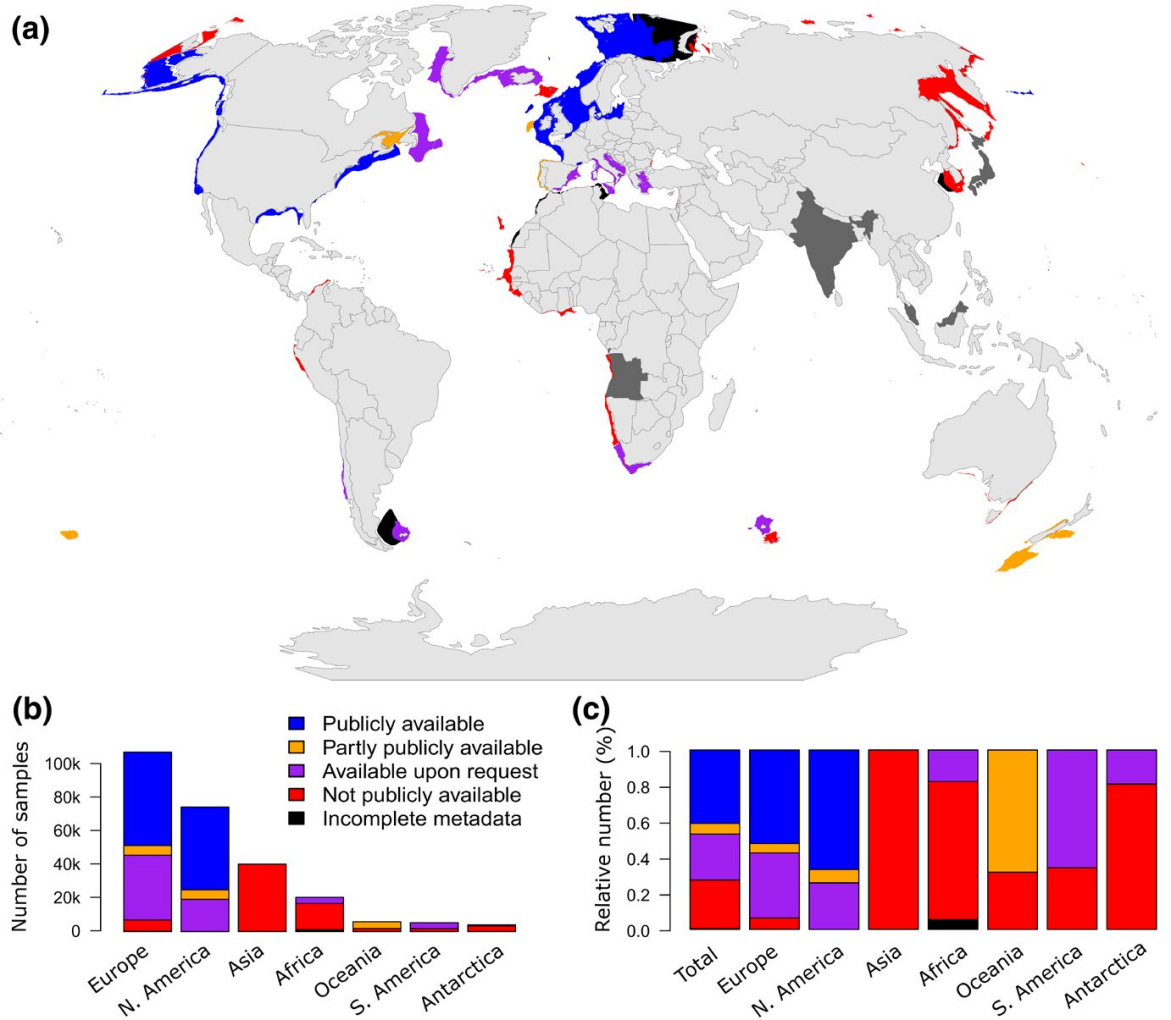
Worldwide availability of bottom trawl surveys, classified as follows: *publicly available* (blue), *partly publicly available* (orange), *available upon request* (purple), *not publicly available* (red), *incomplete metadata* (black) and *unavailable metadata* (dark grey for countries conducting the survey). (a) Location of ongoing scientific bottom trawl surveys, represented by the survey convex hull. Surveys are classified according to their availability. Additional visualizations are available in Supplementary S4. (b) Number of samples for 2001–2019 and availability across continents. (c) Relative availability of samples across continents

The survey metadata built on knowledge of existing survey collections (https://datras.ices.dk/, https://oceanadapt.rutgers.edu/, https://james‐thorson.shinyapps.io/FishViz/) and studies using aggregated surveys (Beukhof et al., 2019; Branch et al., 2010; Gislason et al., 2020; Pecuchet et al., 2017; Pinsky et al., 2013; Trenkel et al., 2019). We sent a standardized query to established and identified contacts of surveys from national fisheries institutes, particularly where geographical gaps were identified (South America, Africa, Asia, and Oceania). We acknowledge that despite rigorous querying, some surveys might still be missing. For each survey, based on haul coordinates, we estimated the spatial area covered using an alpha‐convex hull method (Pateiro López & Rodriguez‐Casal, 2019), with a volume shape set to *α* = 1. Overall, available metadata covered 95 surveys across 78 Exclusive Economic Zones (EEZs) and included 283,925 unique geo‐referenced hauls from 2001 to 2019 (Figure 1; Table S1.1) that covered approximately 2,509,000 km^2^.

### 2.2 Surveys cover productive and fished continental shelves

We compared the spatial extent of the surveys with: (a) the area covered by productive continental shelves, (b) the area fished by bottom trawlers, and (c) fisheries catch. We used global depth data (GEBCO https://www.gebco.net/), chlorophyll‐*a* concentration data (GlobColour GSM, 2005–2015; Maritorena et al., 2010), and bottom trawl fishing data from Global Fishing Watch for the years 2013 to 2016 (Kroodsma et al., 2018). Data were aggregated on a grid (0.04° × 0.04°). High concentrations of chlorophyll‐*a* in continental seas is one of the main factors forming the basis for high fisheries productivity (Stock et al., 2017). Therefore, to define productive shelf sites that could be monitored by bottom trawl surveys, we assigned depth and chlorophyll‐*a* thresholds: a cell was considered a productive shelf site if its depth was 30–500 m—the main depth range targeted by bottom trawl surveys—and if its chlorophyll‐*a* concentration was higher than 0.5 mg/m^3^ (alternative thresholds are shown in Supplementary S3). A cell was counted as fished if more than one trawling activity was detected in the period 2013–2016. We then compared cells with the survey convex hulls to compute the global proportion of productive shelves and fished areas covered by surveys. We estimated that the surveys cover 62% of continental productive seas and 54% of coastal bottom trawl fished areas (Figures S3.3 and S3.5).

To estimate the fisheries productive areas covered by the surveys, we used global marine fisheries landing data for 2000–2014 (Watson, 2017). Landings data were averaged on the time‐period in each 30 min spatial grid cell to estimate an average fisheries production estimate (in tons/year). We computed the ratio of total fisheries landings (tons/year) from areas within the survey areas to the total global landings (tons/year). We estimated that the surveys cover an area that is responsible for 18% of the global fisheries production (Figure S3.6). We repeated the same methodology to estimate the coverage of demersal fisheries production and found that surveys cover an area that represents 20% of demersal fisheries production (Figure S3.7). Both of these ratios are low, likely because the collection of bottom trawl surveys only partly covers demersal communities around the global oceans. A number of productive areas are missing, including tropical seas in Central, South America, and Asia. Second, our collection does not represent many other exploited marine species such as pelagic fish. Pelagic fish monitoring programs exist but were beyond the scope of this study.

### 2.3 Criteria for data accessibility

The survey metadata were classified based on their relative degree of accessibility, using the following classification criteria:

● *Publicly available*: data for all years and species sampled were available in a public repository
● *Partly publicly available*: data only for some years, or only for some species were available in a public repository and access to full data is possible upon request
● *Available upon request*: data are not publicly available but access to data is possible upon request. This category was assigned if at least one person not affiliated to the institution that owns the data obtained the full raw data via request
● *Not publicly available*: when the data, to the best of our knowledge, were not publicly available, access to data is not possible upon request, but access to metadata is possible upon request
● *Incomplete metadata*: when the data were not publicly available and we received access to partial survey metadata via request, or were reconstructed from the literature
● *Unavailable metadata*: when we were aware of ongoing surveys but did not receive access to the metadata, and/or were unable to reconstruct the metadata from the literature

### 2.4 Global status of availability

Among all collected surveys, species abundance/biomass data from 41% of the survey hauls are *publicly available*, while an additional 31% of the surveys are *partly publicly available* or *available upon request* (Figure 1). The remaining 28% of the surveys are classified as *not publicly available* or have *incomplete metadata* and are therefore not available. While species range shifts in response to climate change have occurred across a broad range of aquatic organisms worldwide (Lenoir et al., 2020; Pecl et al., 2017; Poloczanska et al., 2013), most marine studies are concentrated in the northern hemisphere with a majority of surveys located in the North Atlantic and northeast Pacific. This can be explained by the geographical coverage of surveys in the southern hemisphere, which is considerably more restricted and includes almost exclusively not publicly available data, except for South Africa, Chile, New Zealand, Falkland Islands and Kerguelen Islands (classified as *partly publicly available* and *available upon request*, Figure 1a,b). Lower transnational collaboration within Regional Fisheries Management Organizations (RMFOs) in the southern hemisphere may explain this difference in availability. While our international review of the coverage and accessibility of scientific surveys shows that surveys are regularly conducted across continental shelf seas worldwide (78 EEZs), a vast majority of the publicly available data are located in Europe and North America (Figure 1c).

### 2.5 Need for improving data availability

The dominance of northern hemisphere climate change studies has been specifically criticized (Feeley et al., 2017; Lenoir et al., 2020; Lenoir & Svenning, 2015; Richardson et al., 2012). The under‐representation of tropical seas, polar areas, and southern hemisphere studies may mislead our understanding of demersal communities' response to global change. The non‐availability of data can be driven by lack of human and/or logistical resources and capacity to maintain data management systems, or by institutional incentives controlling data access. Furthermore, the difficulty and inability to obtain even metadata from established contacts of current, known surveys, might illustrate that the location of sampling is considered as sensitive, likely from a political and economic perspective. We provide here the most exhaustive assessment of ongoing bottom trawl surveys metadata around the world, and we provide information on who owns these data and where they can be requested, aiming to enhance data sharing (Box 1).

**Applying open science principles to bottom trawl survey data**

Open Science is broadly defined as “Open data and content that can be freely used, modified, and shared by anyone for any [ethical] purpose” (http://opendefinition.org/), and is more specifically described by six main principles (see Gallagher et al., 2020 for a general description). Following is a summary of advances towards Open Science and challenges regarding the use of bottom trawl surveys.

1. **Ensure ethical use of shared information**
  It is crucial that the push towards open science recognizes the value and human side of information. Nations and communities, particularly those that have been historically exploited must be able to benefit from their own data and be able to control their own information to minimize potential abuse. Open science must ensure that open data do not enable an opportunistic fishing company to exploit a nation's or community's resources. While open science can promote transparent science and understanding, it is essential that any use of open data give priority, proper credit, acknowledgement and potentially compensation to those who collected the information, paid for collection and recognize the nation where the data were collected. Access to data from economically stressed nations may require some type of compensation to ensure the data continue to be collected and made available.
2. **Improve knowledge on existing trawl surveys (“Open resources”)**
  Knowledge about the existence of a survey and about the essential course of actions to request and access the survey data can be a challenge. This could be facilitated through a network or a platform where scientists can share such relevant information. Regional platforms currently exist in some areas: Western Africa (http://www.projet‐istam.org/; http://pescao‐demerstem.org/), Southeast Asia (Garces et al., 2006), Europe (https://datras.ices.dk/) and North America (https://oceanadapt.rutgers.edu). Such platforms would ideally improve the visibility of their resources by making their metadata available and easily visible. Here, we established a global network for open resources regarding bottom trawl surveys, where metadata of surveys and contacts or links to access full data are provided (Table S1.1 and https://rfrelat.shinyapps.io/metabts/). The difficulty in obtaining survey metadata and accessing it suggests that challenges remain to create an exhaustive global resource for bottom trawl surveys that can be maintained on the medium and long term.
3. **Improve the accessibility and availability of surveys (“Open data”)**
  An evaluation of the accessibility and availability of surveys is necessary to enhance further open data science. We assessed that 59% of the samples collected are not publicly available to varying degrees (Figure 1). The network created here greatly improves the visibility of surveys, by presenting their metadata. Further, the availability of data can only be improved by changing the way we share scientific information, for instance by publishing data (Costello et al., 2013) and ensuring quality‐controlled use of data. Several bottom trawl surveys are published online, but the most recent years are not always included, or links to access the data are not always maintained, e.g., the Norwegian surveys (Djupevåg, 2018), the southern Gulf of St Lawrence survey (Swain et al., 2016), Mauritania (Kidé et al., 2015), southeast Asian surveys (Garces et al., 2006). Ensuring online publication of data are updated and maintained is key, as is done for other repositories (e.g., DATRAS from the International Council for the Exploration of the Sea https://datras.ices.dk/). Existing platforms that enable online data publication, however, may not always allow updating or involve peer‐review of the data (e.g., PANGAEA https://www.pangaea.de/ and DRYAD https://datadryad.org/stash). To ensure data are available beyond a single report or publication, a dedicated, sustainable, long‐term management strategy is required with dedicated personal. The data repositories mentioned above that update and maintain their information all have dedicated programs and resources to ensure the data are available.
4. **Improve the visibility of the expertise on surveys (“Open source” and “Open methods”)**
  To ensure appropriate use of the data, it is highly important that survey protocols, reports, and common practices are shared together with the raw survey data. Furthermore, providing example code to clean the data or to combine data from multiple surveys can ensure the appropriate use of data. Such types of open source and open access methods have been developed in recent years, mostly for Europe and North America (for instance https://oceanadapt.rutgers.edu/; https://james‐thorson.github.io// for codes; https://datras.ices.dk/ and Moriarty et al. 2017 for codes and published reports). However, such documentation and tools need to be available and easy to find beyond Europe and North America, as well as in multiple languages. Together with the survey metadata information, we started gathering such information (see Table S1.1).

There are many documented cases where disagreements regarding fishing rights have led to serious international conflicts (GuÐmundsson, 2006; Spijkers & Boonstra, 2017). Improved science regarding range shifts across regional boundaries, their impacts on fisheries and fishing communities (Pinsky & Fogarty, 2012; Shackell et al., 2016; Young et al., 2019), as well as political and regulation landscapes (Cheung et al., 2012; Dubik et al., 2019; McIlgorm et al., 2010), could lead to better planning for contingencies regarding climate‐driven distribution shifts. This would provide scientific information to design adaptation and management measures that anticipate potential international conflicts. We therefore argue that financial or political incentives should be identified to better share the existing data, and develop good data management systems (Fenichel & Skelly, 2015). However, benefits from sharing data are diffuse, while their costs (in terms of lost publication opportunities for local teams) are concentrated (Fenichel & Skelly, 2015), and this leads to the well‐known “concentrated‐diffuse'' mechanism for policy failure. Embargo periods could help alleviate some of these issues. This type of policy failure can be partly overcome by concentrating scattered incentives, either by providing multilateral forums where many scientists can jointly benefit from data sharing (e.g., North Pacific Marine Science Organization, https://meetings.pices.int/, International Council for the Exploration of the Sea, https://www.ices.dk/, RFMOs) or by bilateral data sharing agreements (Hammer & Hoel, 2012). The movement towards publicly available and accessible data in science can lead to a lack of recognition of the source, devaluation of essential investments such as data collection, preparation, and curation (Fenichel & Skelly, 2015). As a result, it remains hard to enhance public availability of data. Publishing data, following FAIR principles (Findable, Accessible, Interoperable and Reusable, https://www.go‐fair.org/fair‐principles/) as well as open science principles, could ultimately increase the visibility of surveys but will face the challenge of thoughtfully using the data by prioritizing the need to give credit to the data providers (Costello et al., 2013; Box 1).

## 3 TRANSBOUNDARY SPECIES RANGE COVERED BY SURVEYS

Studies quantifying species range shifts often focus on a specific region covered by one or multiple surveys that might not fully cover the species’ native range (Albouy et al., 2012; Dulvy et al., 2008; Morley et al., 2018; Perry, 2005). In fact, species’ ranges may extend well beyond the monitored area and the resulting range shifts may be misrepresented by the survey(s). Demersal fish habitats are often only partially covered by surveys, particularly since surveys are designed to sample soft bottoms on primarily shallow continental shelves, hence excluding hard bottoms and reefs. Most of the surveys are limited by depth, sampling the continental shelves but rarely the slopes at greater depths. Moreover, ecosystems beyond national jurisdiction are often excluded, which is problematic in case of straddling stocks. To assess the percentage of species range covered by current surveys, and to evaluate the probability of species range shifts to occur beyond surveyed areas, we compared the habitat from species distribution models to the areas covered by the surveys.

To estimate the extent to which existing surveys cover species distribution range, we selected the top three demersal species with the highest commercial catch in each of 19 FAO fishing areas from FishStats (FAO, 2017; http://www.fao.org/fishery/statistics/software/fishstat/), defined as the average catch over 2001–2019. We ended up studying 37 demersal species (with some species covering several FAO areas). For the commercial species identified, we downloaded the native range from AquaMaps (Kaschner et al., 2016), which shows the probability of occurrence of each species on a 0.5° × 0.5° grid. We used the modeled native range and considered it as the “true” habitat and species range. The habitat in AquaMaps is based on publicly available global occurrence data and expert judgment on species environmental niches. Even though AquaMaps may sometimes misrepresent species ranges because of poor occurrence data and lack of knowledge for some species (O’Hara et al., 2017), the ranges of the selected commercial species are generally well documented. The preferred habitat data layer of each species for the analysis was defined as all locations from the AquaMaps habitat maps where the probability of occurrence was higher than 0.5 (alternative thresholds are shown in Supplementary S5).

**FIGURE 2:**
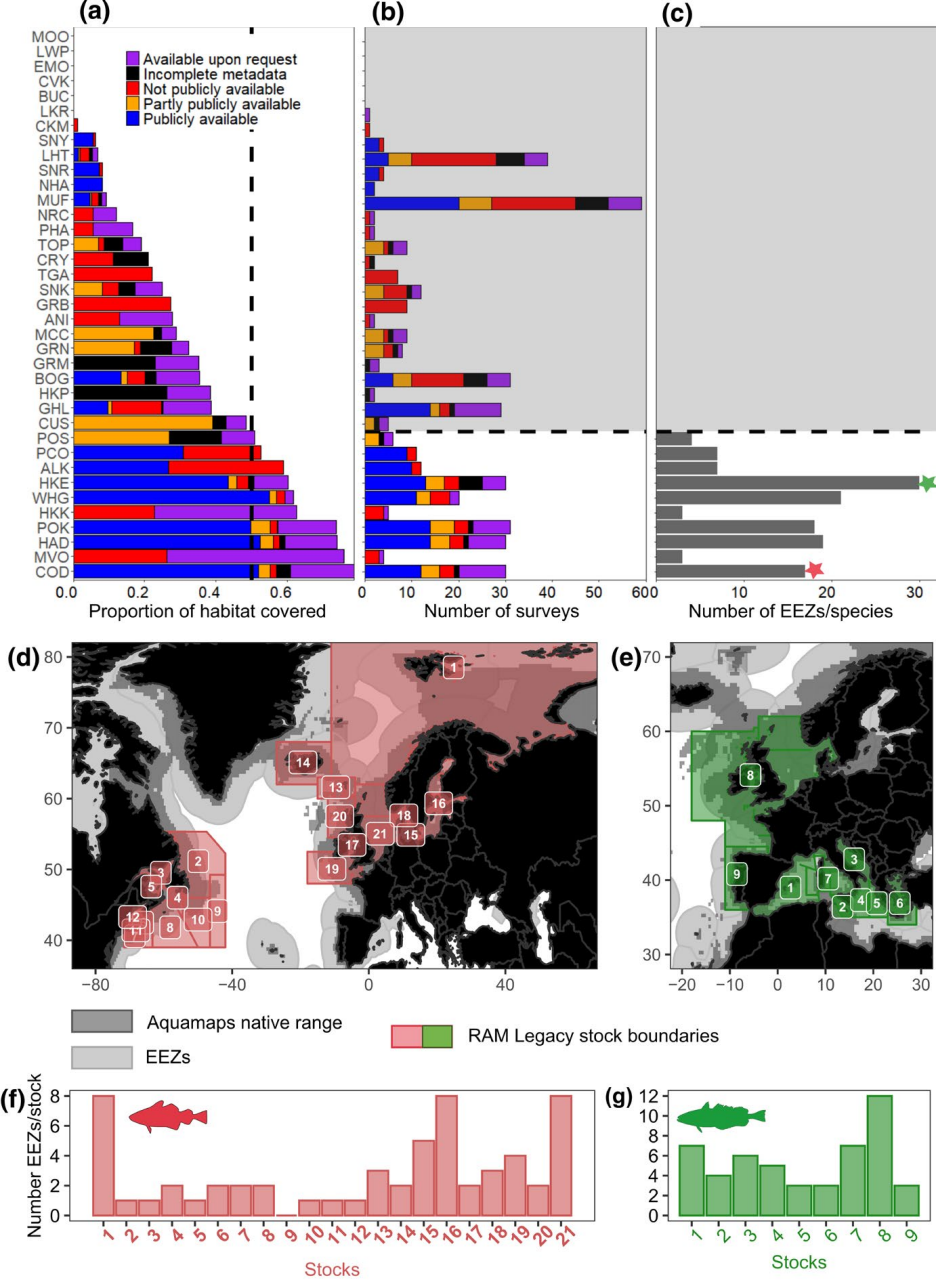
Transboundary commercial species covered by surveys. Main commercial demersal species are identified by the ASFIS 3‐letter codes and the corresponding coverage by the surveys: (a), proportion of species AquaMaps habitat covered by the surveys (the vertical dotted line indicates 50% of range covered); (b), number of surveys behind the proportion covered (species for which less than 50% of range covered are shaded, colors indicate the availability status attributed to each survey); and (c), number of Exclusive Economic Zones (EEZs) covered by the species range based on AquaMaps. Stars on (c) indicate Atlantic cod (pink) and European hake (green). Corresponding Latin names to species are available in Table S5.1. For Atlantic cod (d) and European hake (e), maps display the EEZs covered by AquaMaps species range, as well as fish stocks spatial boundaries as recorded in the RAM Legacy Stock Boundary Database. The bottom panel further indicates the number of EEZs covered by each Atlantic cod and European hake stock ((f) and (g), respectively). Figures in (d–g) show the number of EEZs overlapped by each stock. Note than stock delineation in some of these stocks (e.g., stock number 1 for hake) is under current research and may change in the near future

Next, we calculated the percentage of cells from the AquaMaps species habitat area covered by the survey footprints, showing the overlap between the two. We also included the availability status of surveys. We demonstrate that no combination of available surveys covers the entire range of any single species (Figure 2a). Nevertheless, for about a quarter of the species considered, existing surveys cover more than 50% of the species habitats, up to a maximum of 79% for Atlantic cod (*Gadus morhua*). However, even for these well‐surveyed species, the surveys are sometimes not available (MVO, *Lophius vomerinus* and HKK, *Merluccius capensis* in Southeast Atlantic and South Africa) or only a part are publicly available (PCO, *Gadus macrocephalus* and ALK, *Gadus chalcogrammus* in the North Pacific). We computed the number of surveys that overlapped with the species native range and show that the number needed to cover at least 50% of the main commercial species habitat is highly variable (from 4 to 31, average of 18, Figure 2b) and depends on the areas covered by each survey. The restricted spatial extent of some surveys conducted in Europe explains why more than 15 surveys may be needed to cover 50% of a specific species range. AquaMaps may be a poor proxy for the habitat of some species, where the habitat suitability is under/over‐estimated. We expect that this would particularly affect the proportion of native habitat covered by surveys, but not much the number of surveys required to cover the range. For instance, if the distribution is wrongly projected in unoccupied areas by the species (e.g., offshore), the estimated number of surveys is less likely to change. Even if potentially inaccurate for some species, this large open source of data demonstrates the need for standardizing surveys and developing tools to combine data from different surveys.

In addition to being covered by multiple surveys, species can also be spread over multiple EEZs, and are in this case transboundary. To assess whether species are transboundary, we calculated how many EEZs are covered by each of the 10 species mostly covered by surveys (Figure 2c), using species’ predicted distribution from AquaMaps as the species range. All 10 species are transboundary species and 50% of them are spread over more than 10 EEZs. Importantly, these species are not managed at a species level, but at a population (or stock) level. Climate change is affecting marine life at multiple levels from species, to stock complexes containing populations, to single fished stocks (Free et al., 2019). To assess fisheries stocks that may be transboundary, we used the RAM Legacy Stock Boundary Database (Free et al., 2019), focusing on the available spatial boundaries of 21 Atlantic cod stocks (*Gadus morhua*) and 9 European hake stocks (*Merluccius merluccius*) as case studies (Figure 2d–g; see Supplementary S6 for methodological details). Atlantic cod is spread over more than 18 EEZs (Figure 2c), and its stocks are transboundary as well (14 out of 21 stocks are spread over more than 1 EEZ, Figure 2d,f). Similarly, European hake range covers 30 EEZs, and all stocks are transboundary (Figure 2e,g). We note, however, that this database of stock boundaries is under revision, and so these numbers should be taken as illustrative but not definitive. It is critical to improve our knowledge of stock delimitations and population complexity, which differ across systems/species, and can be altered by climate change (Kerr et al., 2017). Most species in the North Atlantic such as cod and hake are already managed within management areas representing multiple countries. However, the management of transboundary shifting species still requires specific adaptation, starting with an accurate assessment of species geographical redistribution over management areas and EEZs by combining multiple regional surveys.

## 4 TRACKING SPECIES DENSITIES ACROSS MANAGEMENT AREAS

We have shown that demersal fish ranges and habitats are not fully covered by bottom trawl surveys, which may be particularly problematic when species and fisheries stocks are transboundary. However, the capacity to track such transboundary species throughout their range critically depends on the ability to combine surveys from multiple sources and regions. In cases where data are available and gathered from different sources, formatting, language differences and lack of user expertise on the survey itself may limit the ability to use the data appropriately. For instance, information on units, haul duration or swept area estimates are sometimes lacking, limiting the combined use of multiple independent surveys. In addition, differences in gear, sampling designs, species identification procedures and catchability across and within surveys may bias perceptions of species distribution and regional changes in abundance. In order to standardize processing of such data, we recommend improving the availability of survey documentation, including explanations of survey methodology and associated coding that can be freely applied to clean, standardize, and combine surveys (Box 1). Making expert knowledge easily accessible will facilitate studies combining multiple surveys (see for instance Moriarty et al., 2017).

### 4.1 A case study to combine surveys across regions

**FIGURE 3:**
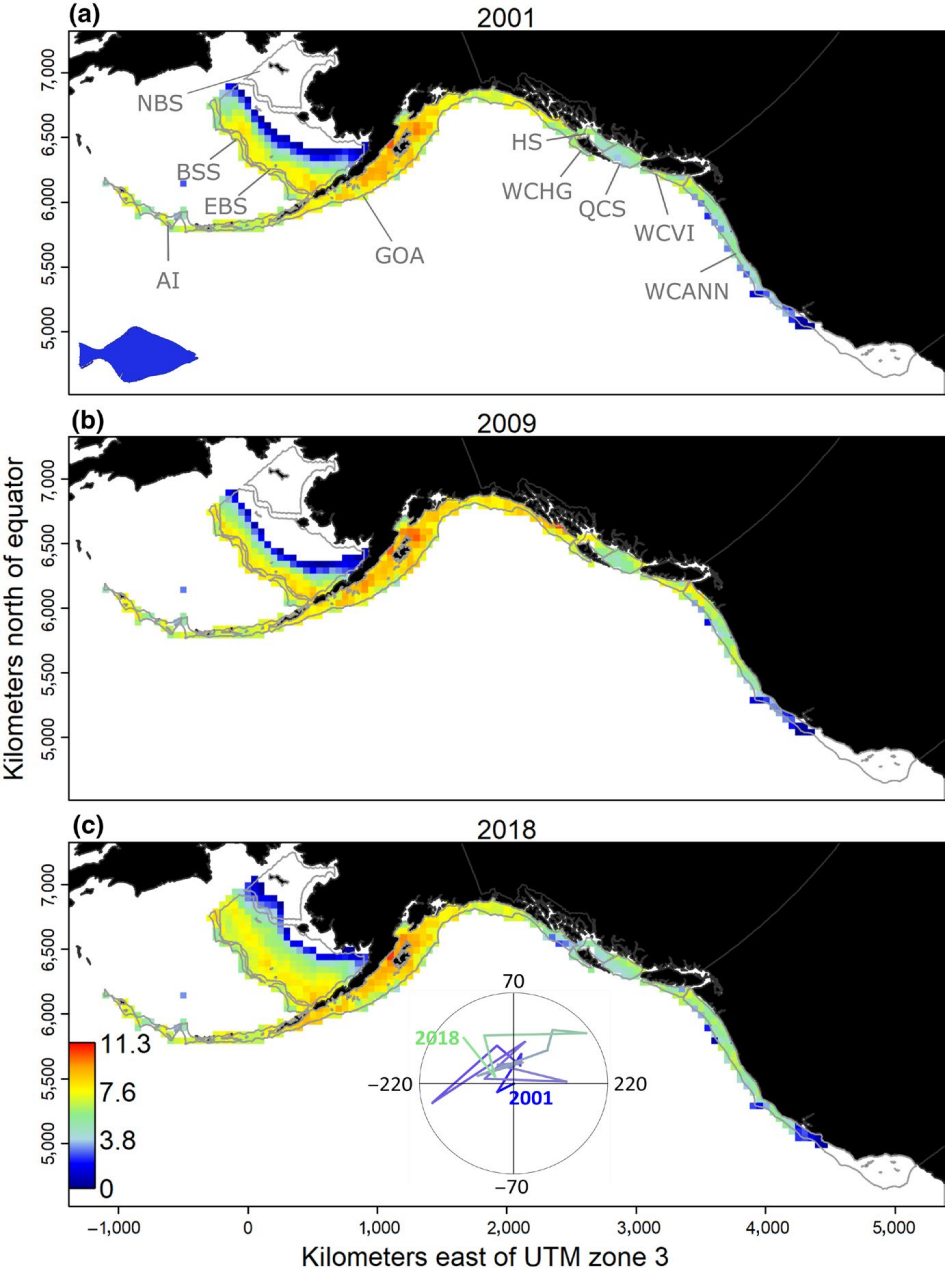
Density estimates for arrowtooth flounder (*Atheresthes stomias*) along the northeastern Pacific coast containing contiguous sampling data from multiple surveys in log(kg/km^2^) using regional bottom trawl surveys: West Coast US (WCANN), West Coast Vancouver Island (WCVI), Hecate Strait (HS), West Coast Haida Gwaii (WCHG), Queen Charlotte (WCS), Gulf of Alaska (GOA), eastern Bering Sea (EBS), northern Bering Sea (NBS), Aleutian Islands (AI), and Bering Sea slope (BSS). Polygon contours represent the different surveys, as indicated by the corresponding codes. Densities are presented for 3 years: (a) 2001, (b) 2009, and (c) 2018. Only densities higher than 0.1% of the maximum were selected to clearly differentiate areas occupied by the species (color‐coded) from those mostly unoccupied (white). The inset in the bottom panel shows the change in the center of gravity through time, where longitude and latitude for 2001 are (0;0)

We used arrowtooth flounder (*Atheresthes stomias*) to illustrate how to combine survey data across multiple regions when tracking and investigating population‐scale range shifts in species distribution. Arrowtooth flounder is a widespread and ecologically important predator in the northeast Pacific (Aydin & Mueter, 2007), monitored and assessed by 10 distinct but contiguous surveys across the region from the California Current to the Bering Sea between 2001 and 2018, conducted by the United States and Canada (Supplementary S7; Figure 3a). To predict densities within the entire survey domain, we fit a spatio‐temporal Poisson‐link delta‐gamma model (Thorson, 2017) to biomass data from each survey using the R‐package VAST (Vector Autoregressive Spatio‐Temporal, Thorson, 2019b; Thorson & Barnett, 2017). This model has the advantage of interpolating density across time and space within the survey domain when survey data are lacking in a given area or time step (Supplementary S7). We assumed that each survey has identical gear performance (i.e., catches the same proportion of individuals within the area‐swept by bottom trawl gear). The validated model shows that the highest densities of arrowtooth flounder are observed in the center of distribution within the Gulf of Alaska (Figure 3). However, densities have recently increased in the eastern Bering Sea and the distribution has shifted inshore and northward. Simultaneously, its distribution has slightly moved southward in the California Current. Despite this expansion at both ends of its range, the centroid of the population shows a net change northward by 40 km in less than 20 years (Figure 3c).

### 4.2 Improving the management of transboundary species

The use and coordination of multiple surveys is needed to monitor commercially important species distributions that extend beyond a singular survey. Our case study and other recent studies show that statistical models can reconstruct species densities across surveys, even when they follow non‐overlapping designs (Dolder et al., 2018; Ono et al., 2018; Selden et al., 2020; Thorson et al., 2016). This type of modeling can appropriately quantify the changes of densities through time and across regions by correcting for unbalanced sampling (O’Leary et al., 2020; Thorson, 2019b; Thorson & Barnett, 2017). The multiple‐survey approach—applied here to the arrowtooth flounder—is applicable for many other wide‐ranging species, including commercially important transboundary species such as Greenland halibut (Wheeland & Morgan, 2019), Pacific cod (Drinkwater, 2005), and Atlantic cod (Morley et al., 2018; Spies et al., 2020). For each of these species, combining surveys will require initial research to determine the most appropriate methods to account for differences in catchability between surveys, species, and sites (Fraser et al., 2007; Moriarty et al., 2020; Thorson, 2019b; Walker et al., 2017). This could be done by using regression‐discontinuity‐designs to estimate catchability ratios for surveys that are contiguous, but not overlapping. Furthermore, ongoing efforts to standardize national surveys will also help to combine surveys: Russia and Norway started a joint ecosystem‐wide survey in the Barents Sea (Eriksen et al., 2018), and Mediterranean countries ensure consistent sampling protocol across EU regions of the Mediterranean Sea through the MEDITS program (Spedicato et al., 2020). Our knowledge of species redistribution across surveys will clearly benefit from long‐term consistent surveys, when combined and modeled appropriately.

Long‐term global monitoring datasets are essential to develop transboundary science, and offer opportunities to improve the management and conservation of migrating transboundary species (Box 2). Under global change, migrating species create a potential for economic and political conflicts (Mendenhall et al., 2020), and may lead to species overexploitation or collapse in the case of lack of adaptation and cooperation (Miller & Munro, 2004; Oremus et al., 2020; Pershing et al., 2015; Pinsky et al., 2018; Vosooghi, 2019). The 1982 United Nations Convention on the Law of the Sea (UNCLOS) provides the legal framework for international obligations towards safeguarding marine resources. Migratory and transboundary stocks are principally managed by RFMOs (Aqorau et al., 2018; Miller & Munro, 2004; VanderZwaag et al., 2017), or conservation‐related initiatives such as the Global Transboundary Conservation Network (http://www.tbpa.net/). Still, these organizations need to explicitly consider governance in the context of climate change (Oremus et al., 2020; Pentz et al., 2018; VanderZwaag et al., 2017). While building a common understanding of status and trends is a key first step towards transboundary cooperation (Pinsky et al., 2018), international governance will require global geopolitical flexibility and the establishment of transnational agreements (Miller et al., 2013; Scheffers & Pecl, 2019), which need to be supported by cross‐boundary open science (Boxes 1 and 2). The adaptation of management and policy is essential for the sustainability and conservation of shared resources, but are often motivated by economic and cultural values rather than ecological considerations (Scheffers & Pecl, 2019). Clear and transparent scientific evidence of geographical distributions can help to incorporate species biogeography into political decisions (for instance, in the case of fisheries quotas policy). We developed a framework (Box 2) where scientifically supported transboundary governance will avoid overexploitation, conflicts about newly or historically shared resources, and conservation of vulnerable species. Global research efforts (as in this study) can be further developed to identify vulnerable species, for instance by applying a trait‐based approach (Albouy et al., 2020; Payne et al., 2020). Identifying vulnerable relocating or shifting species and building the capacity for adaptation can both aid responsive and efficient governance of species affected by climate change.

**Towards a framework for transboundary management and conservation**

1. **Agreement of Survey Data and Analyses**. All parties must agree on data, information, current abundance and spatial footprint of the fisheries stock and species of interest
  - *Find the surveys covering the species range*. The first key task is to establish which surveys cover the native range of the species of interest. The next task is to find and access surveys corresponding to that species native range. A list of existing surveys, their time coverage and available samples are available here for demersal species (see Table S1.1). If surveys are not publicly available, one can use the network of contacts published in this paper and/or establish bilateral/multilateral agreements to gain access to the survey data. Metadata should also ideally include a list of species recorded in the surveys.
  - *Estimate the change in species density and distribution*. Surveys vary in terms of design, gear, catchability, and sampling methods. Multiple surveys can be combined to estimate species density and reconstruct past temporal changes in spatial distribution. Modeling the change in species distributions can be done with multiple models and needs to take into account survey discrepancies (Dolder et al., 2018; Ono et al., 2018; Selden et al., 2020; Thorson et al., 2016). Sharing information across international boundaries could enable a more complete picture of the distribution of a fish population and reconsideration of the definition of their stock structure.
  - *Forecast changes in densities and distributions*.
    Building on the knowledge of past species distribution change and ecology, forecasting species distribution will enable adaptation to changes in advance (Payne et al., 2017). The spatio‐temporal model described here can also be used to forecast species distributions in the near future (Thorson, 2019a), and thus the spatial scale at which adaptation measures should be applied.
  - *Measure ecosystem impact in local management areas*.
    Climate change enhances the dynamic nature of changes in species abundance and requires fisheries management to adapt, not only directly to the resource, but also to assess the impacts on port infrastructure, fishing fleets/gears and other human activities (Greenan et al., 2019; Pinsky & Fogarty, 2012; Young et al., 2019). The immigration/emigration of species into local areas can lead to substantial changes in community structure and diversity, and may lead other species to outcompete or be outcompeted. By monitoring not only commercial species, but the entire community—as is generally possible with bottom trawl surveys—we can understand ecological changes and inform the conservation of vulnerable species (Pinsky et al., 2020; Scheffers & Pecl, 2019).
2. **Management and cooperation**
  - *International agreements*.
    All parties must create a management agreement for the regulation of the resource that is legally binding, regardless of how the distribution or abundance of the resource might change or not change in the future. Policies could be developed within the agreement to adjust regulations depending on a range of future scenarios such as when stocks move poleward, or decrease/increase in abundance. Preagreements covering a range of options can help reduce future conflicts and reduce the need to renegotiate or abandon the agreement (Hilborn et al., 2001). An important goal of the management agreement is to acknowledge that changes are likely to occur, while recognizing that the specific change is likely unpredictable.
  - *Transboundary cooperation*.
    In the case of transboundary species and distribution over multiple management areas, changes in spatial distribution under climate change and variability may favor/exclude countries or regions (Golden et al., 2016; Oremus et al., 2020). Therefore, some areas will “win” or “lose” and create conflicts and/or lead to species overexploitation (Mendenhall et al., 2020; Pinsky et al., 2018). Building agreements among countries to share resources equitably—or compensate when not possible—is necessary to ensure the sustainability of resources and the dependent human communities (Miller & Munro, 2004). It is essential that all parties perceive benefit from cooperating and remaining within the agreement. In the case of non‐exploited/non‐targeted species, cooperative conservation actions should be established. Such cooperation can only be built with open and transparent science (Box 1) to conserve the species and avoid conflicts.
  - *Regulation and enforcement*.
    To truly implement transboundary management and conservation, the involved parties must develop a method to enforce their agreement. Effective monitoring to gather information on the shared resource and to measure compliance is important. Compensation and/or penalties may also be involved to ensure all parties adhere to the regulations. Side payments are one means of compensation that can take different forms (Miller & Munro, 2004). Direct cash payments are possible, however countries can also share monitoring and research capacity across international boundaries as is done for a number of stocks that straddle Canadian and United States waters (Miller & Munro, 2004). Nations can allow other nations to fish for a specific shared resource in their EEZ as is done between Norway and Russia (FAO, 2002; Hønneland, 2014) or swap quota in a multispecies fishery as has been done in the Baltic Sea (Ranke, 2003). Once again, the goal is to develop an agreement in which all parties perceive benefits to properly manage shared stocks.

## 5 MAINTAINING SURVEYS TO FACE FUTURE CHALLENGES IN THE OCEANS

Regular trawl surveys do not cover the entire continental shelves and the lack of monitoring makes it problematic to track ecosystem change and adapt management and policy to shifting resources. Additional sources of information could be considered to better cover demersal species habitats. Such sources could include fishery‐dependent data (such as observer, landings, vessel report trip data) that are able to report species occurrences and in some cases catch‐per‐unit‐effort. Data derived from the fisheries industry have the potential to: (a) indicate the presence and abundance of species where scientific surveys are not conducted (Hilborn & Walters, 2013); (b) derive abundance estimates and spatial coverage by combining fishery‐dependent and fishery‐independent data (Nielsen et al., 2019; Pennino et al., 2016; Pinto et al., 2018); (c) provide global information on marine species from all types of habitats (for instance, http://www.seaaroundus.org/, used in Pinsky et al., 2018 or Watson, 2017); (d) understand socio‐ecological fisheries systems under shifting resources (Greenan et al., 2019; Pinsky & Fogarty, 2012; Young et al., 2019). Other promising sources of data could be derived from environmental DNA (eDNA; Pikitch, 2018; Salter et al., 2019). eDNA is a non‐invasive and cost‐efficient tool that can adequately detect the presence of fish in bottom waters, notably rare ones, and can potentially estimate relative fish abundances (Afzali et al., 2020; Russo et al., 2020; Salter et al., 2019). This powerful tool is, however, still in its infancy and further development is needed to standardize it against bottom trawl catches before wide implementation (Garlapati et al., 2019). In addition, citizen science initiatives reporting species observed well outside their typical geographic ranges (e.g., the Range Extension Database and Mapping project; Redmap [Pecl et al., 2019] https://www.redmap.org.au/ and the European Alien Species Information; EASIN [Schade et al., 2019]; https://easin.jrc.ec.europa.eu/easin), also add valuable evidence of species range shifts in poorly sampled areas.

Surveys are highly valuable for following marine species redistribution and biodiversity change, but maintaining surveys in a consistent way through time is a challenge as they are costly. However, ecological time series become more informative the longer their timespan, highlighting the need to maintain long‐term monitoring programs (Hughes et al., 2017; Schindler & Hilborn, 2015). The existence of international programs such as the Nansen program (http://www.fao.org/in‐action/eaf‐nansen/en/) is valuable to inform ecosystem‐based management (Bianchi et al., 2000, 2016) and could be expanded. Surveys impact seafloor habitat, benthic communities, and sampled fish (Trenkel et al., 2019) and we should ensure that this kind of monitoring benefits science as much as possible. Surveys must be designed to be as efficient as possible by sharing (meta)data, providing opportunities for innovative uses of the data and improving the economic and ecological efficiency of monitoring. In any case, challenges of sampling marine communities and sharing data need to be overcome to allow scientific assessment and adequate management of shifting marine resources.

## 6 CONCLUSION

The concentration of marine studies in the northern hemisphere profoundly limits not only our ability to track and understand climate change effects and species range shifts, but also our capacity to adapt, mitigate or avoid potential conflicts and socio‐economic consequences that follow. This is particularly important in parts of the developing world where fisheries constitute a primary source of food and livelihood for coastal communities, but information supporting management is often scarce or non‐existent. To alleviate these issues, a coherent framework to monitor, understand, and inform sound and scientifically underpinned management actions to adapt to species range shifts is needed. Our study provides a first step towards creating such a framework by conducting a joint and internationally coordinated synthesis of the global coverage and availability of survey data, and it will be of great assistance to various users aiming to assess and predict the response of marine biodiversity to climate change. Our study has identified important gaps in data availability and accessibility, and suggested ways to make the best use of surveys at hand by combining data from multiple sources to assess species redistributions over multiple management areas. We make a general plea for open science as well as fair and transparent sharing of data. This is needed to support scientific advice on coordinated spatial management actions, allowing us to adapt and prepare for the inevitable ecological and socio‐economic consequences of climate change yet to come.

## CONFLICT OF INTEREST

The authors declare no competing interests.

## AUTHOR CONTRIBUTION

Aurore A. Maureaud, Romain Frelat, Laurene Pecuchet, Nancy Shackell and James T. Thorson designed the project, directed and performed the data collection and analyses. Aurore A. Maureaud led the project and Romain Frelat led the data curation. James T. Thorson produced the code and ran the model to estimate density of species with multiple surveys. Romain Frelat, James T. Thorson and Aurore A. Maureaud produced figures and conducted analyses. All authors have either provided metadata, provided contact lists and/or lists of surveys to help collection of the metadata, conducted metadata requests and/or helped with the interpretation of results and writing the manuscript. Aurore A. Maureaud wrote the first draft of the manuscript and Romain Frelat, Laurene Pecuchet, Nancy Shackell, Bastien Merigot, Malin Pinsky, and James T. Thorson largely contributed to editing and writing. All co‐authors were given the opportunity to revise the manuscript.

## Supporting information

Supplementary Material

Supplementary Material

## Data Availability

Publicly available and partly publicly available (meta)data presented are accessible with the links provided in Supplementary S1, Table S1.1. (Meta)data that are not publicly available cannot be made available in this paper and therefore only a summary is accessible in Supplementary S1 (Table S1.1). All codes and analyses are available on GitHub (https://github.com/AquaAuma/TrawlSurveyMetadata), including the surveys convex hull created with the survey metadata and the R code to reproduce figures and analyses.
