# Supplementary material for: Are we ready to track climate‐driven shifts in marine species across international boundaries? ‐ A global survey of scientific bottom trawl data
Source: Glob Chang Biol. 2020 Nov 10;27(2):220–36. doi: 10.1111/gcb.15404 (PMC7756400; doi:10.1111/gcb.15404)

Boops boops (BOG)

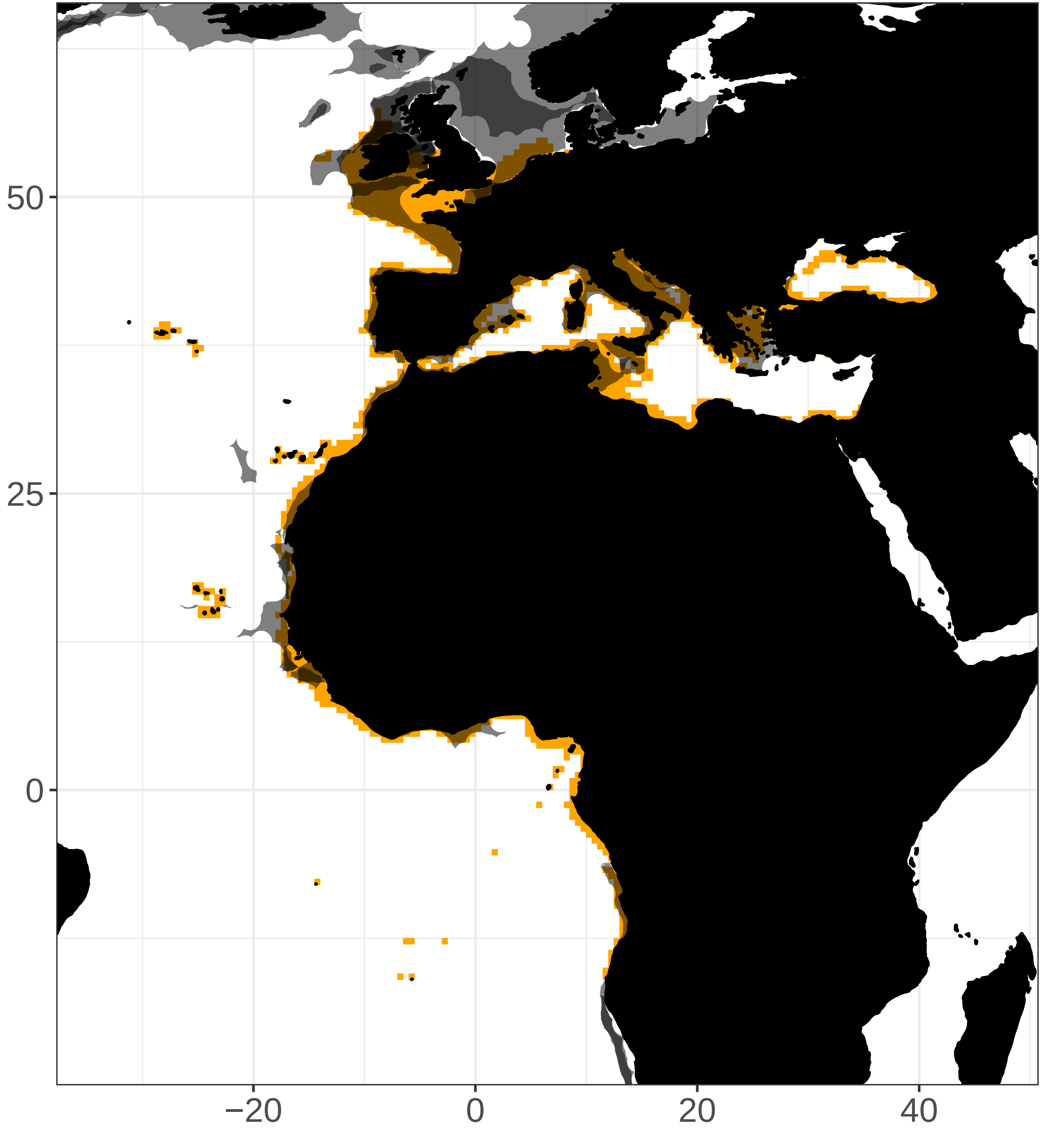

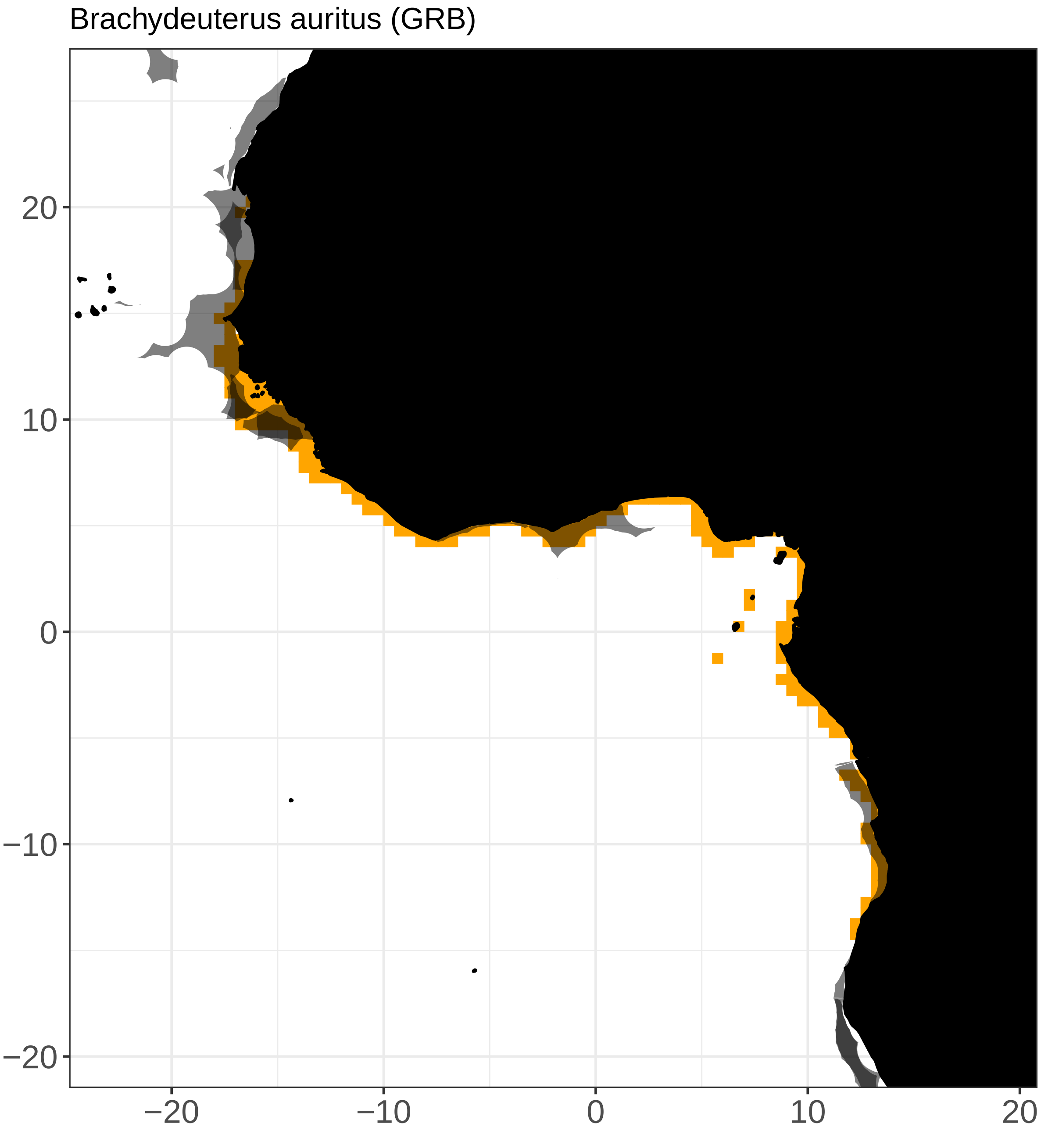
Brachydeuterus auritus (GRB)
20
10
0
−10
−20
−20
−10
0
10
20

Cephalopholis boenak (CVK)

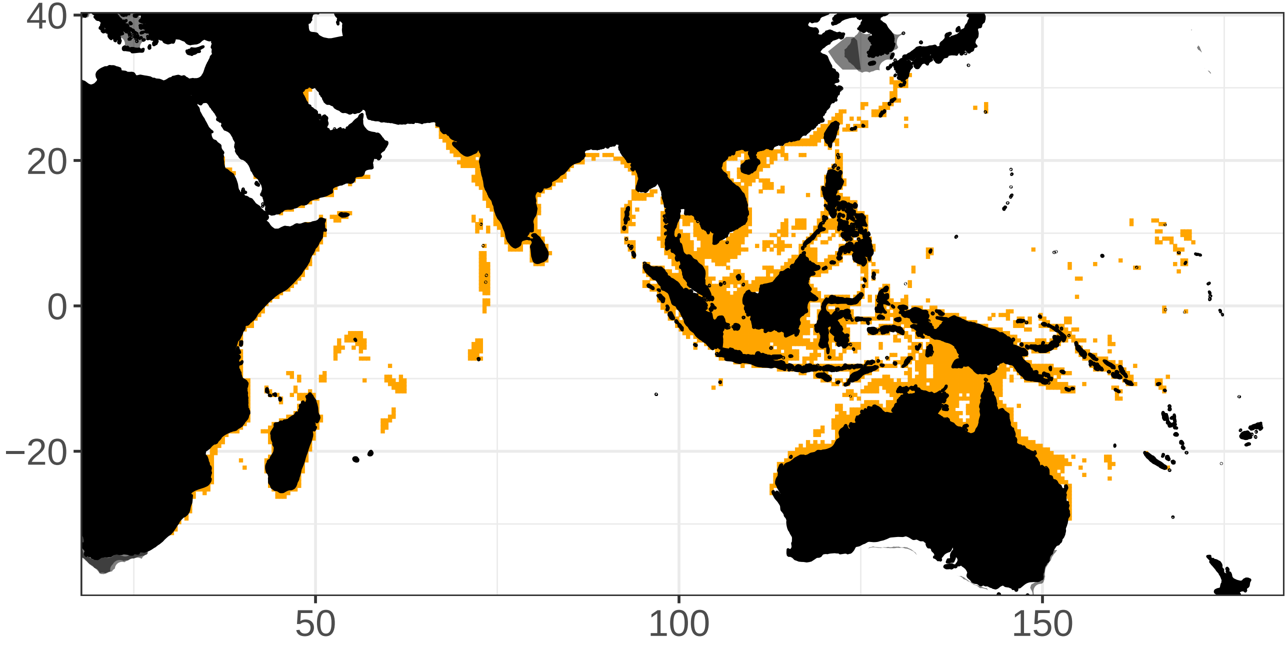

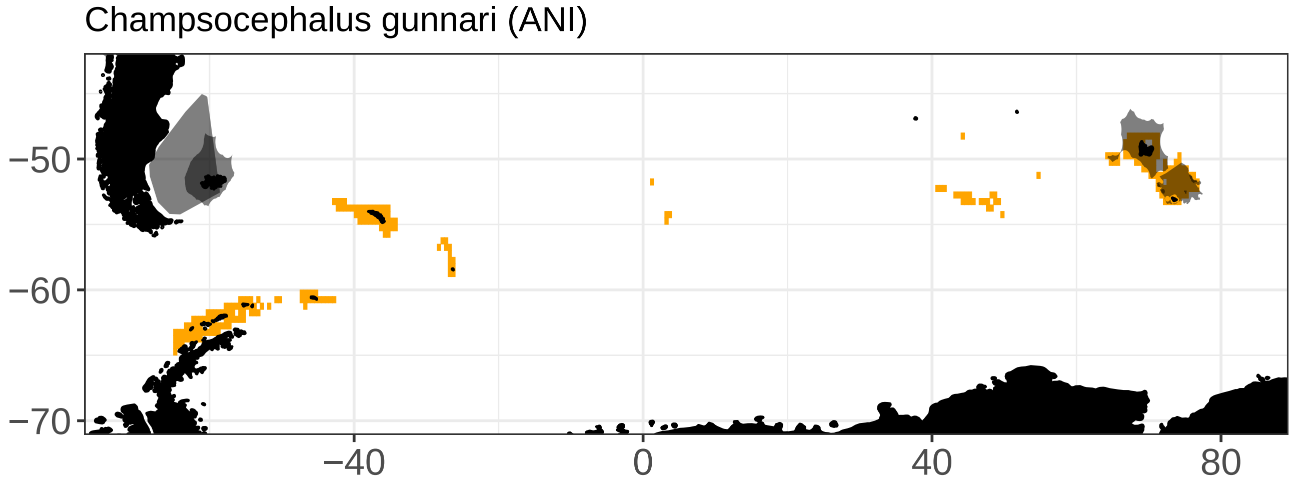

Champsocephalus gunnari (ANI)
−50
−60
−70
−40
0
40
80

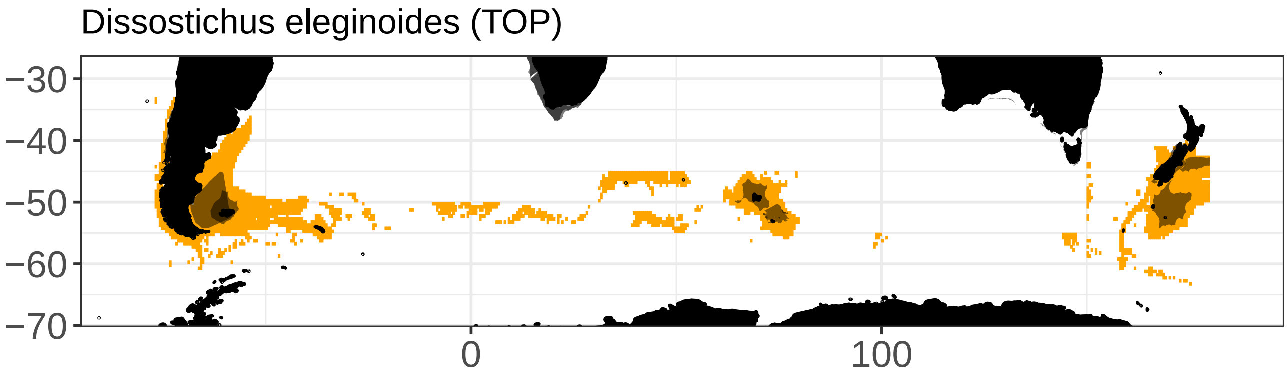

Dissostichus eleginoides (TOP)
−30
−40
−50
−60
−70
0
100

Gadus chalcogrammus (ALK)

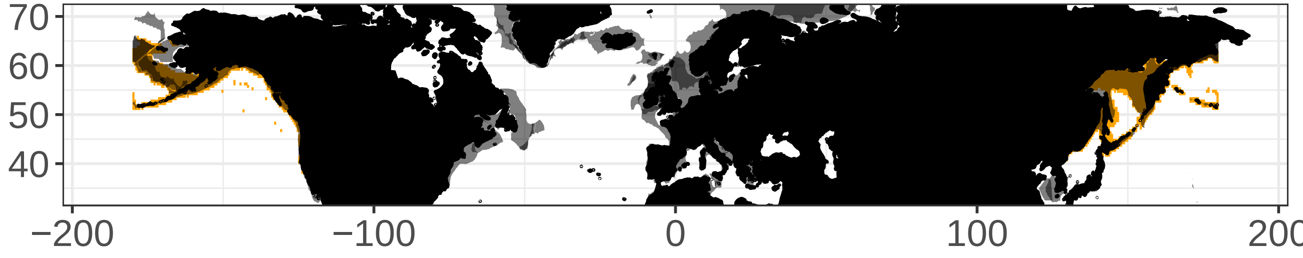

Gadus macrocephalus (PCO)

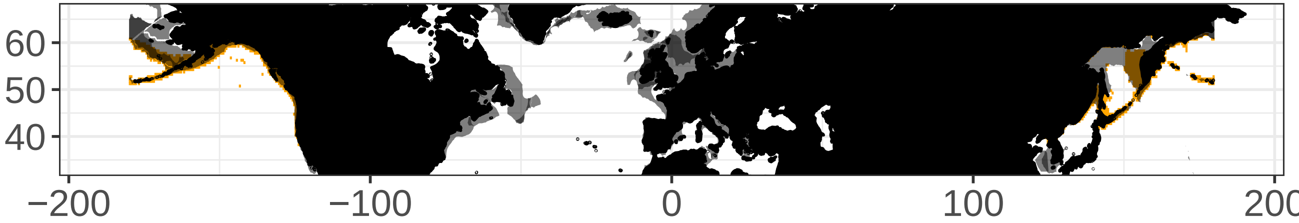

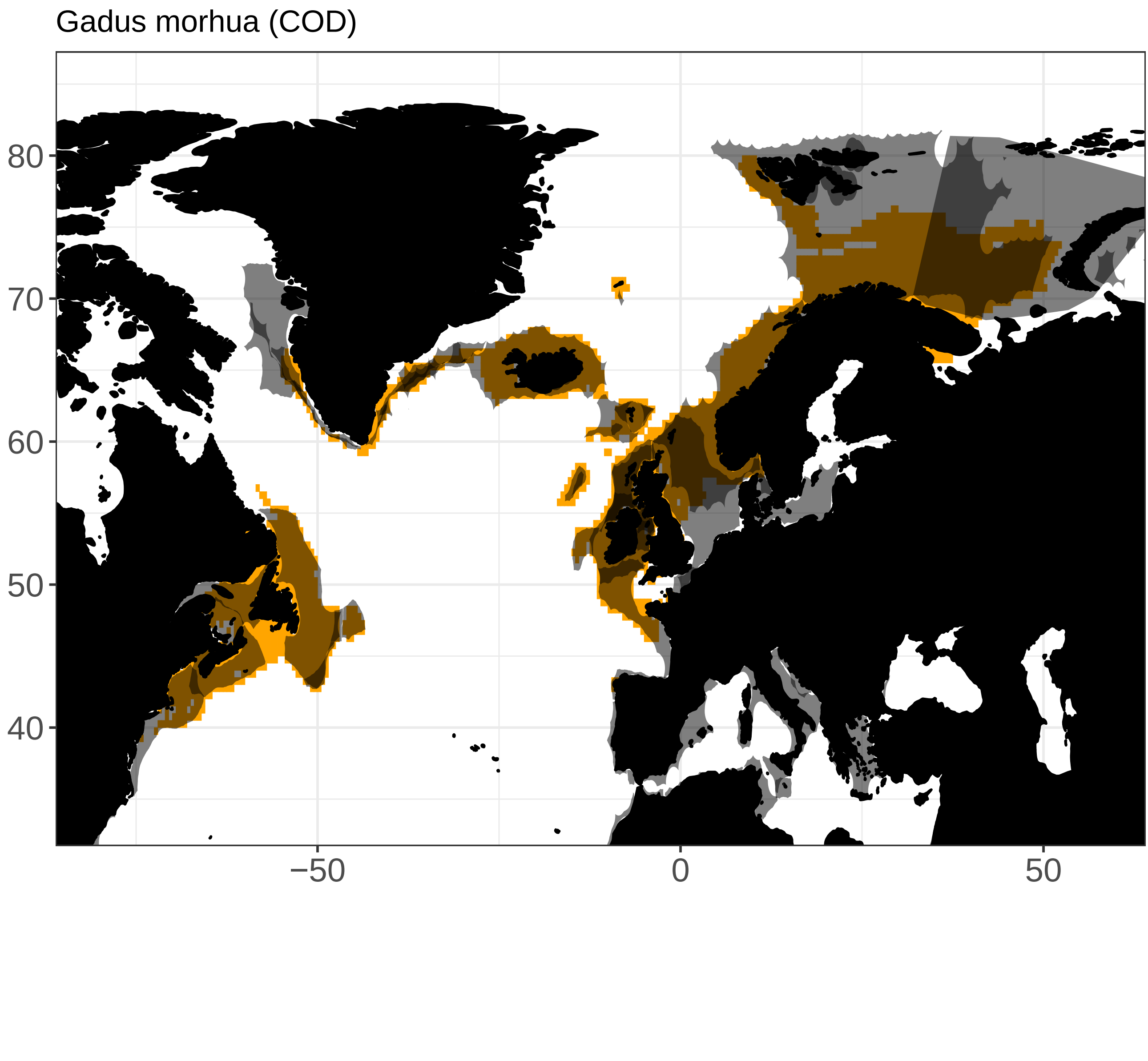
Gadus morhua (COD)
80
70
60
50
40
−50
0
50

Genypterus blacodes (CUS)

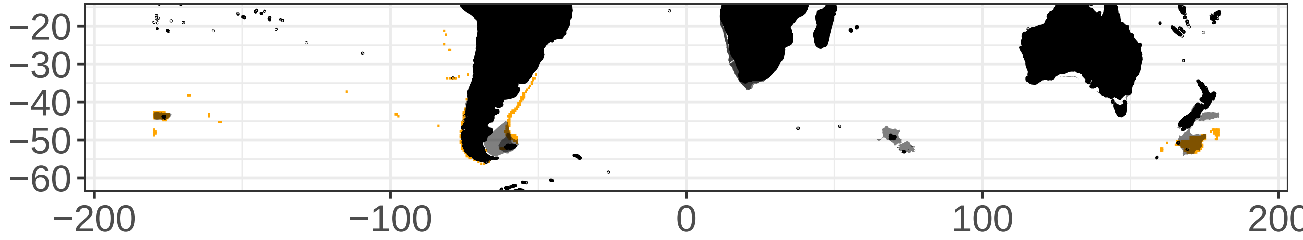

Harpadon nehereus (BUC)

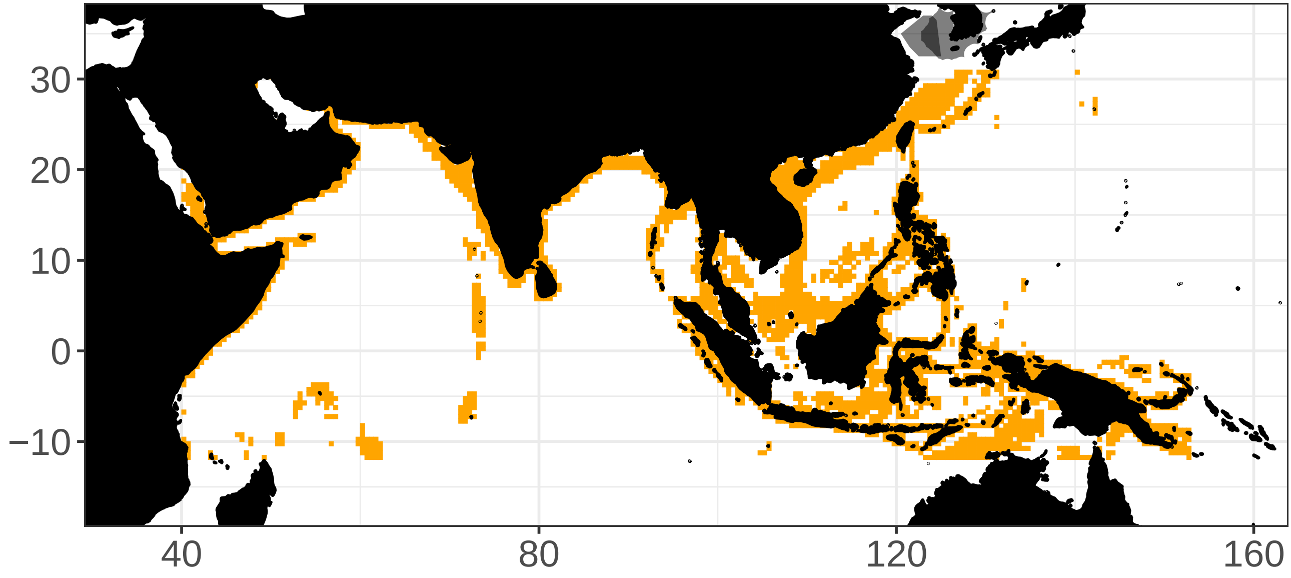

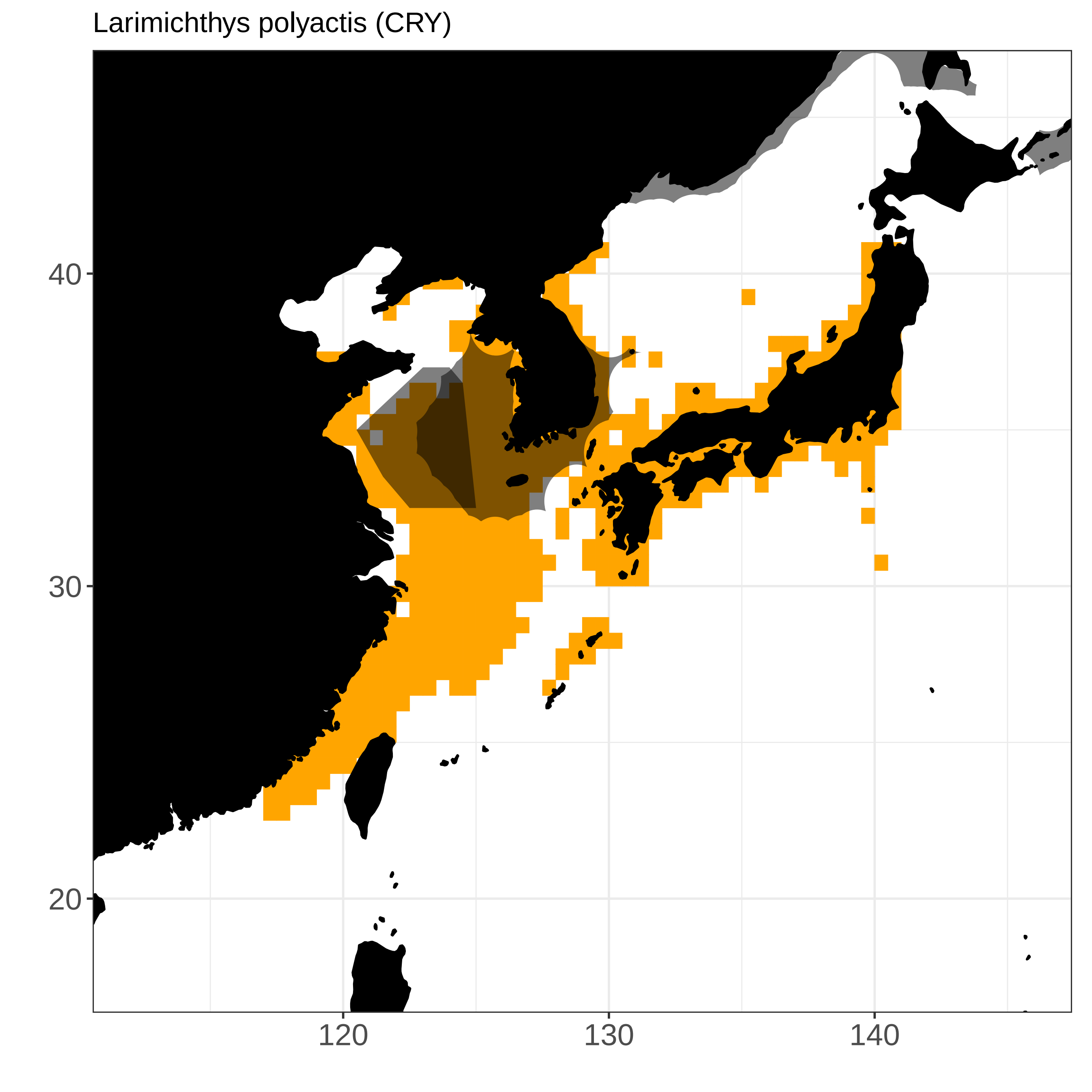
Larimichthys polyactis (CRY)
40
30
20
120
130
140

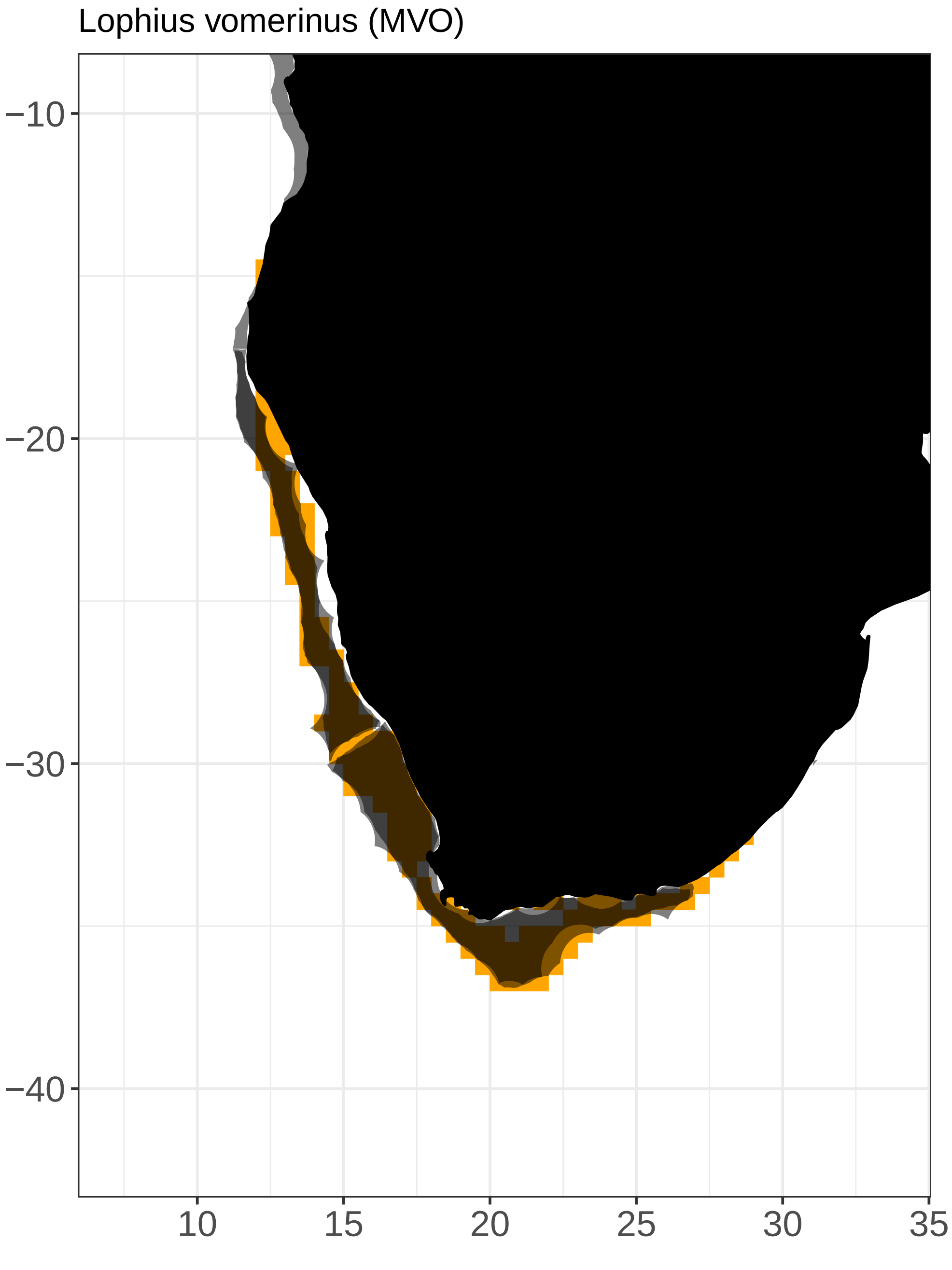
Lophius vomerinus (MVO)
−10
−20
−30
−40
10
15
20
25
30
35

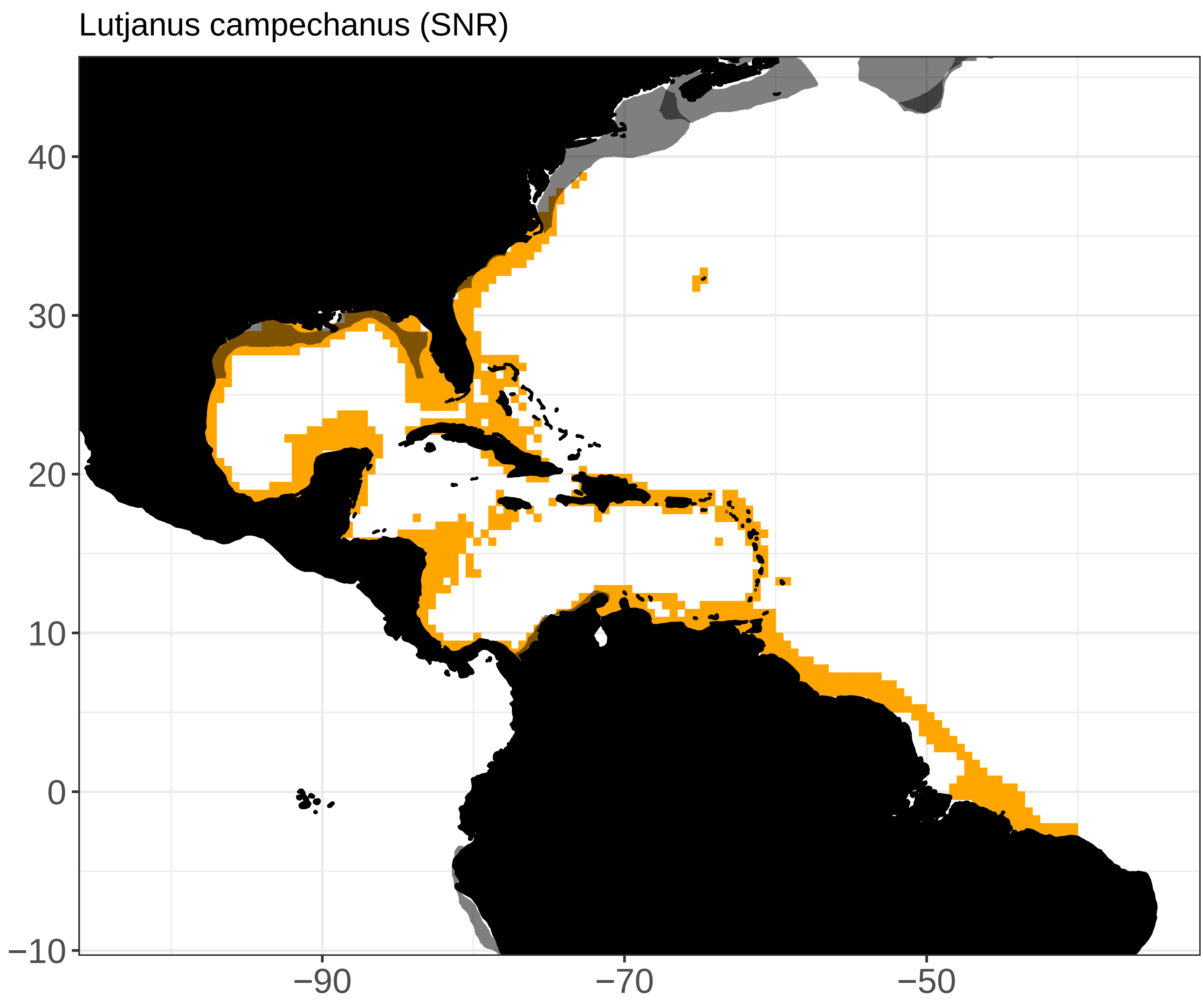
Lutjanus campechanus (SNR)
40
30
20
10
0
−10
−90
−70
−50

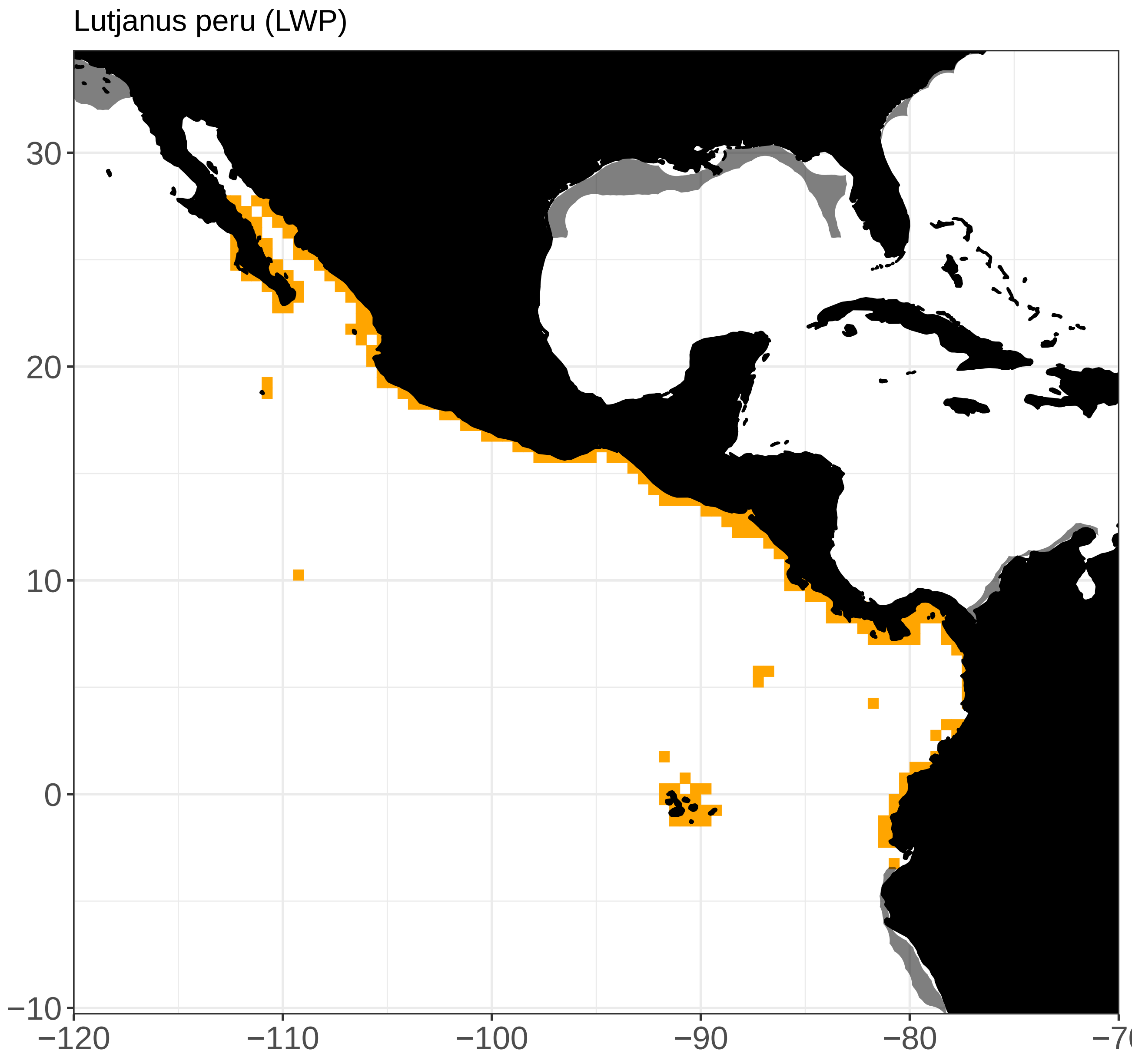
Lutjanus peru (LWP)
30
20
10
0
−10
−120
−110
−100
−90
−80
−70

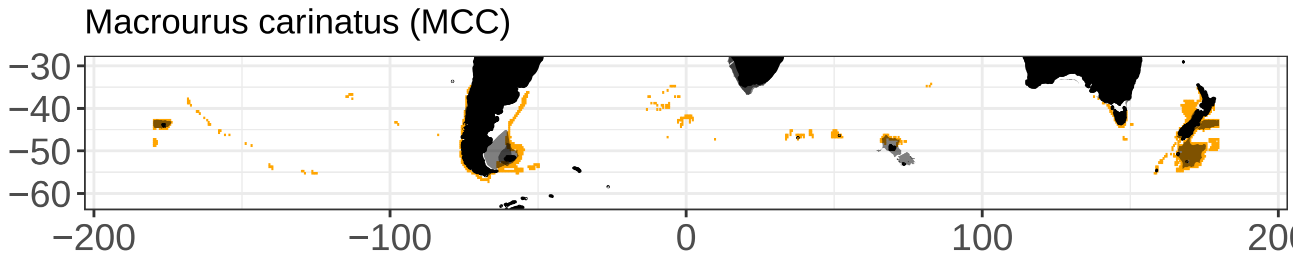
Macrourus carinatus (MCC)
−30
−40
−50
−60
−200
−100
0
100
200

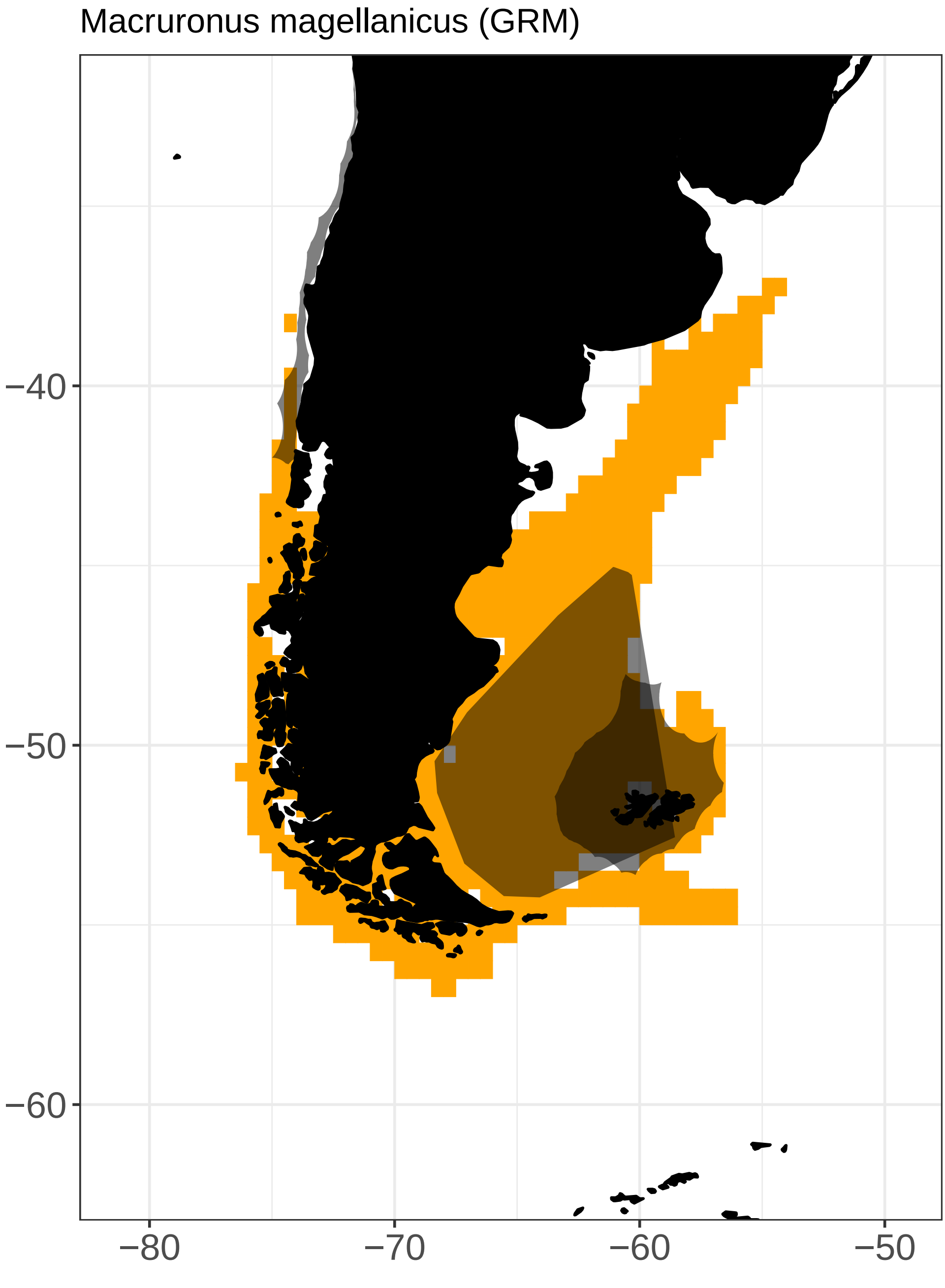
Macruronus magellanicus (GRM)
−40
−50
−60
−80
−70
−60
−50

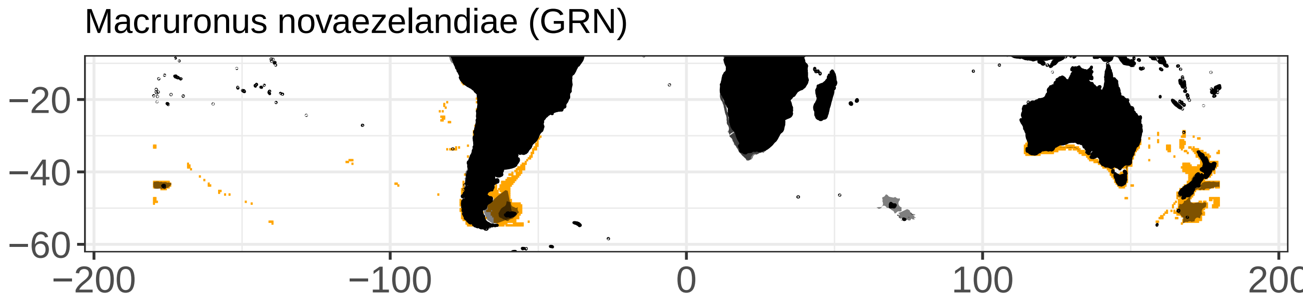
Macruronus novaezelandiae (GRN)
−20
−40
−60
−200
−100
0
100
200

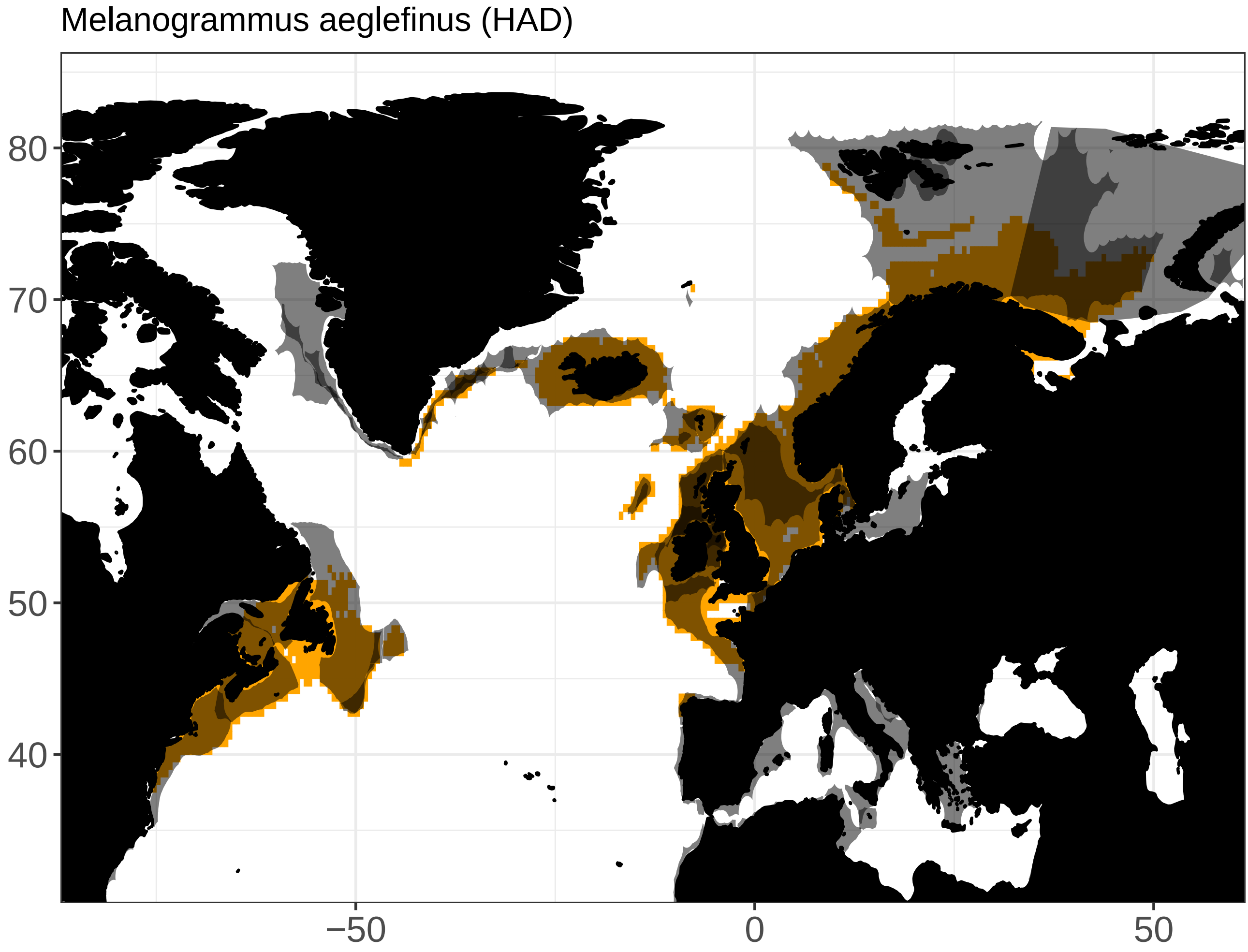
Melanogrammus aeglefinus (HAD)
80
70
60
50
40
−50
0
50

Mene maculata (MOO)

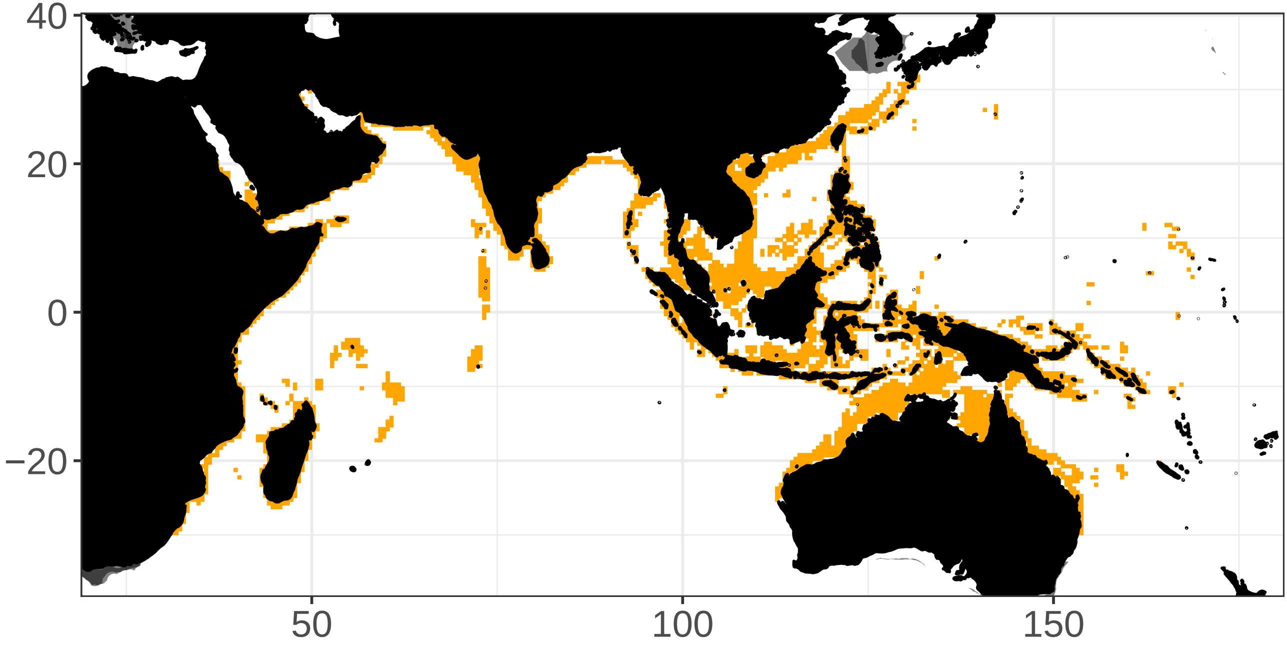

Merlangius merlangus (WHG)

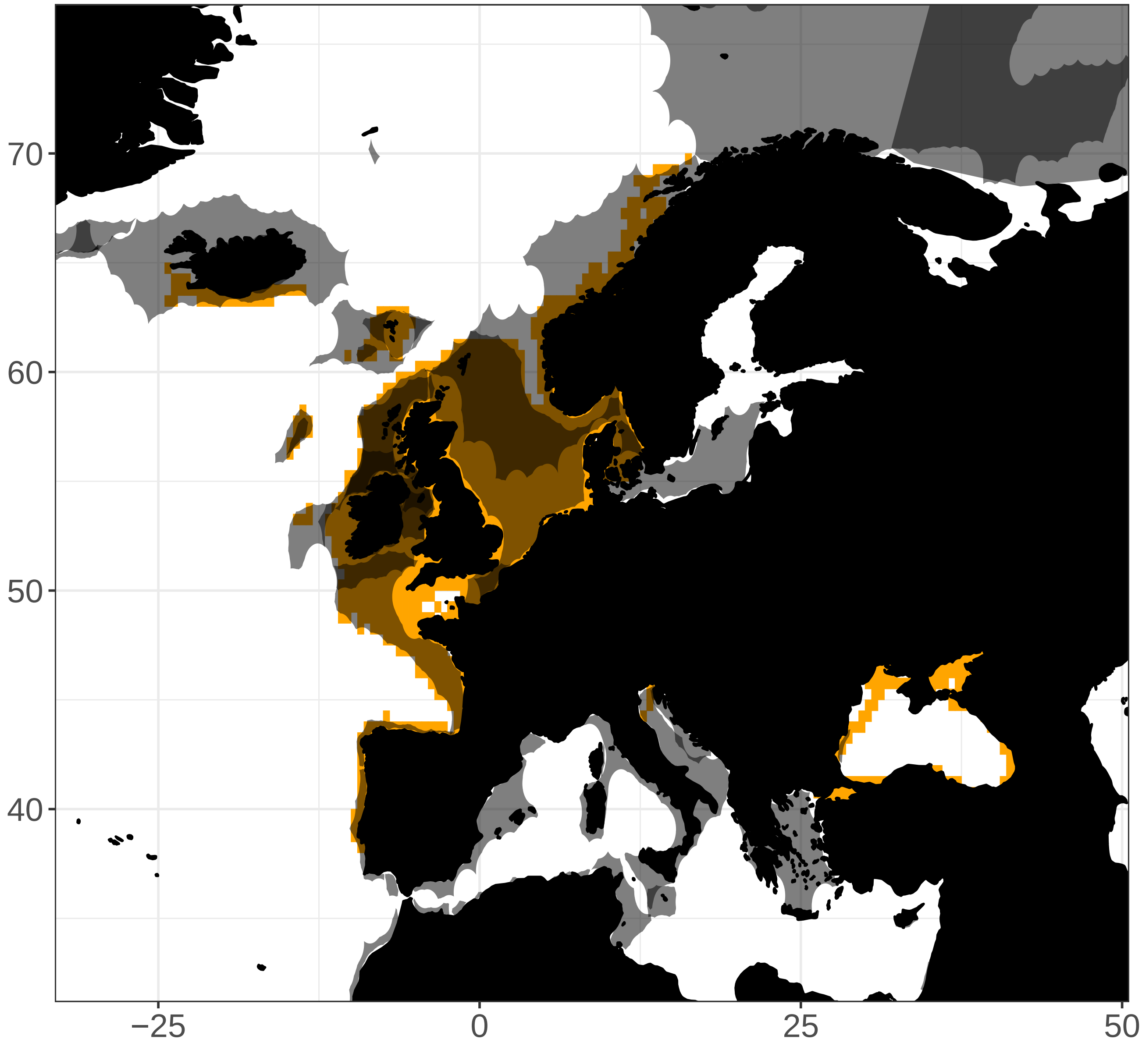

Merluccius capensis (HKK)

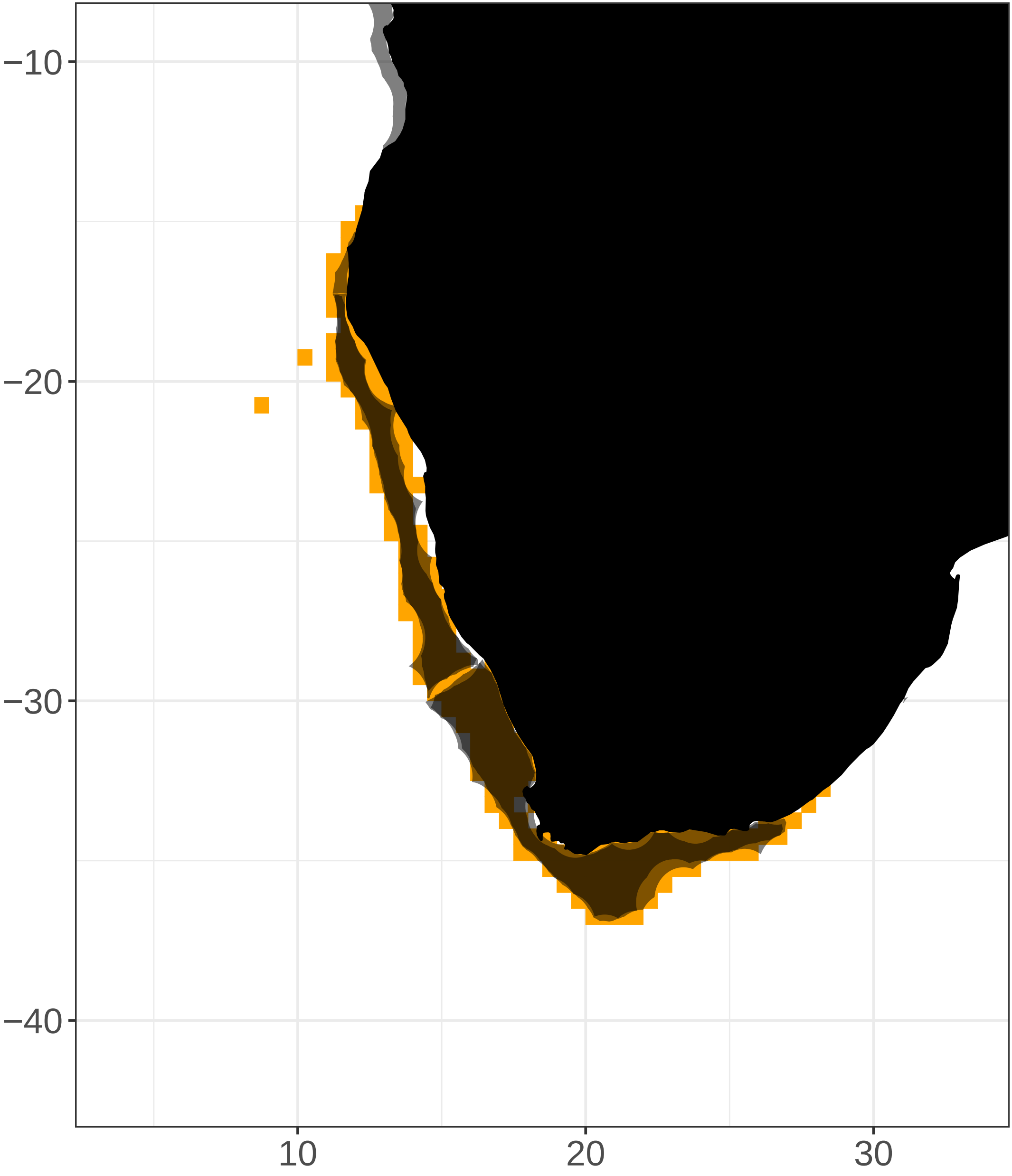

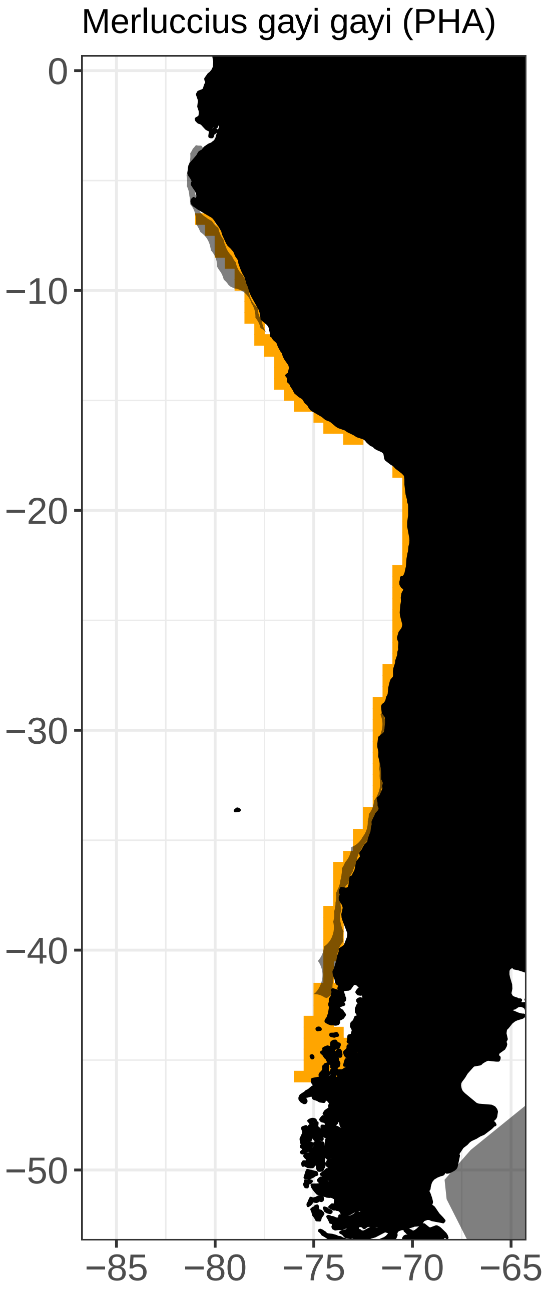

Merluccius gayi gayi (PHA)
0
−10
−20
−30
−40
−50
−85
−80
−75
−70
−65

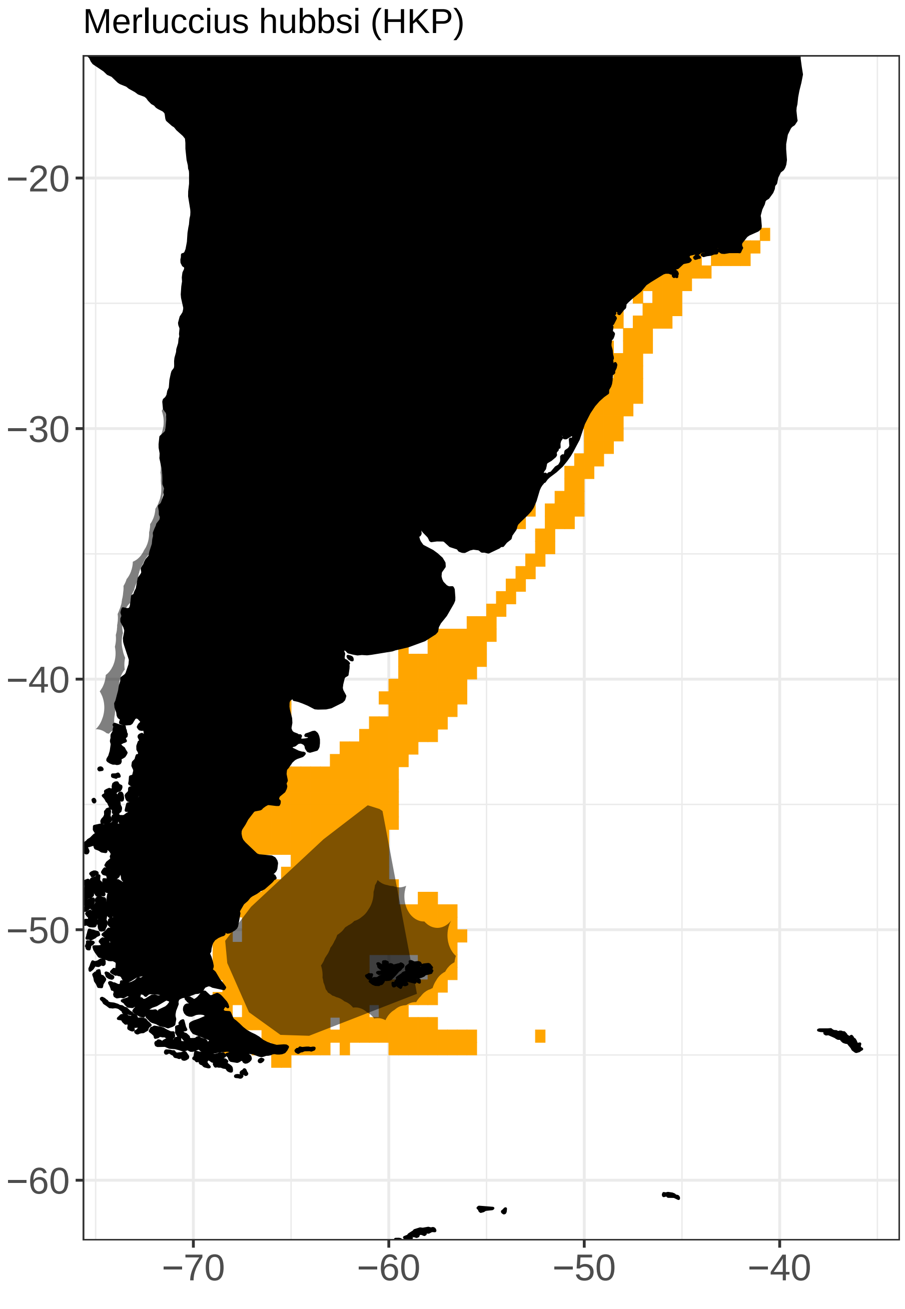
Merluccius hubbsi (HKP)
−20
−30
−40
−50
−60
−70
−60
−50
−40

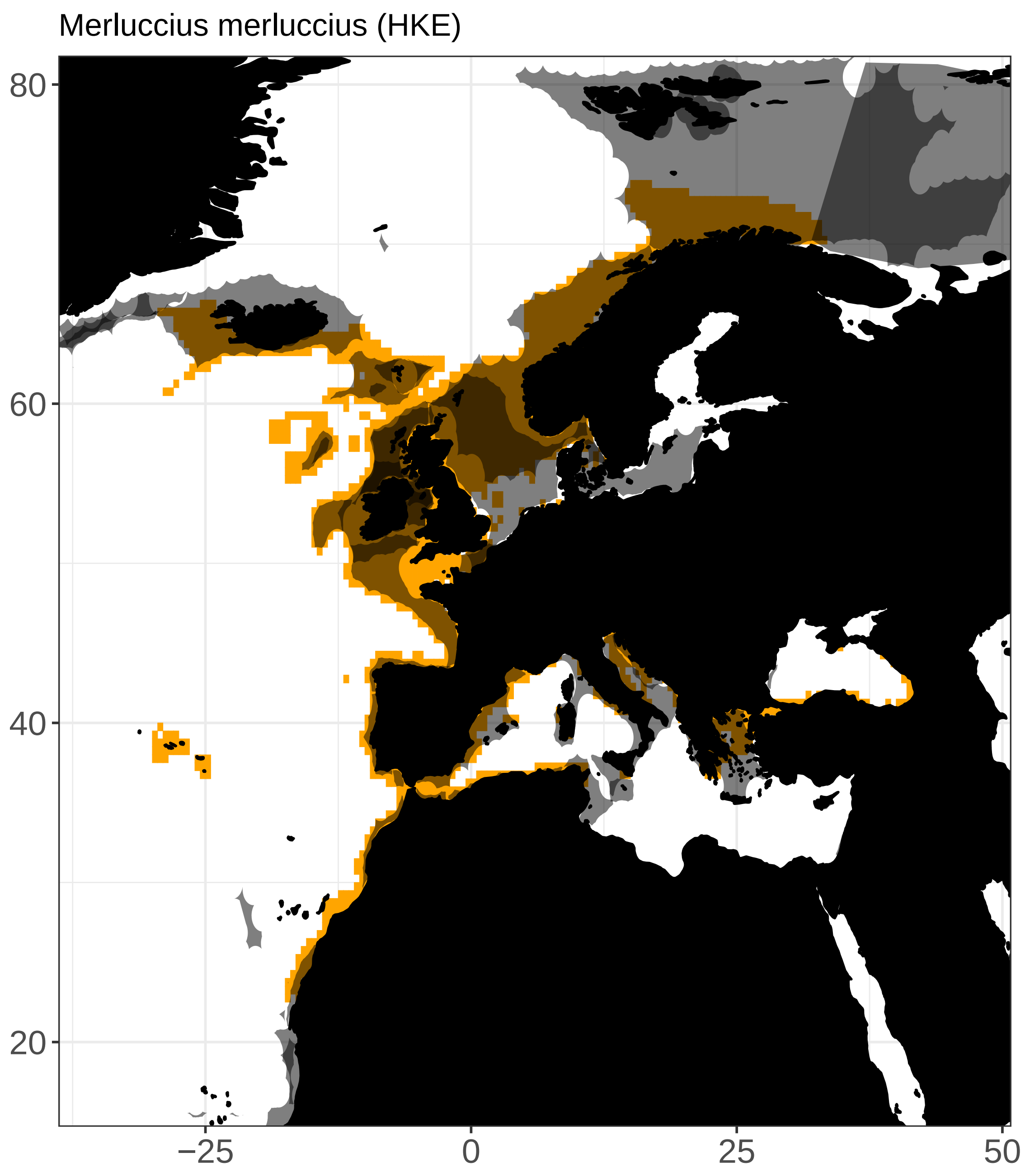
Merluccius merluccius (HKE)
80
60
40
20
−25
0
25
50

Merluccius productus (NHA)

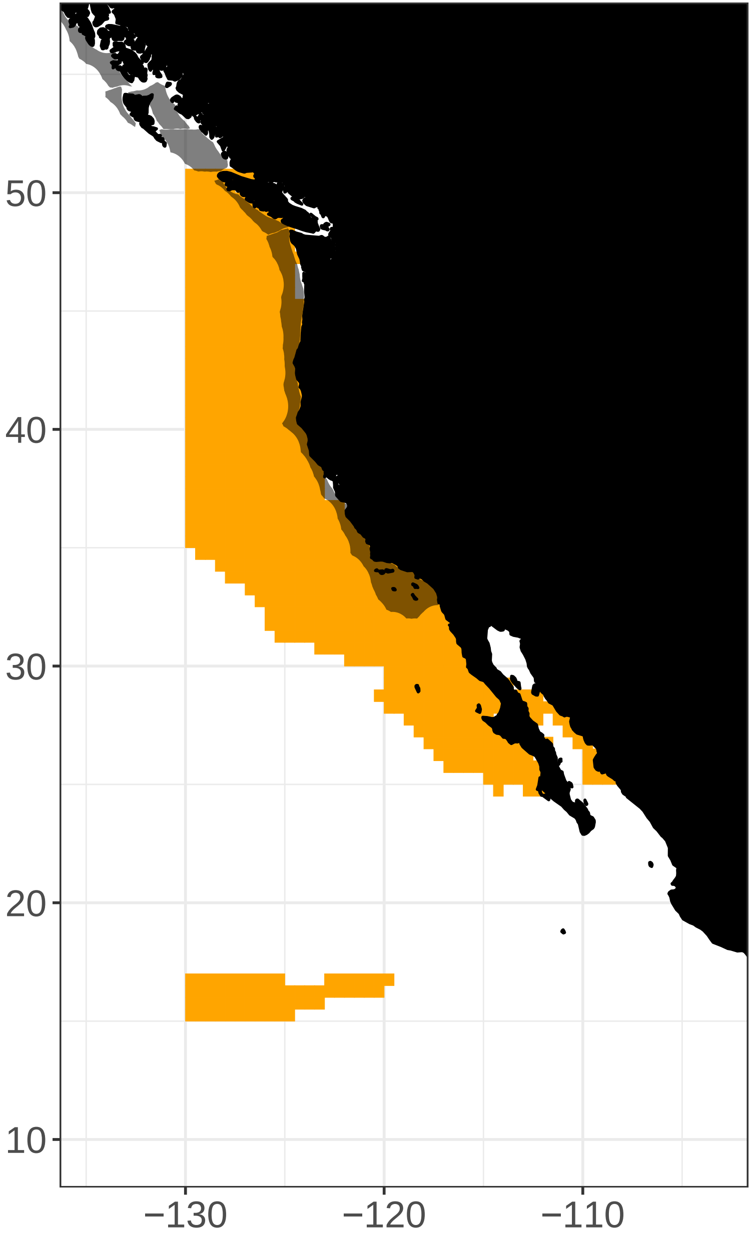

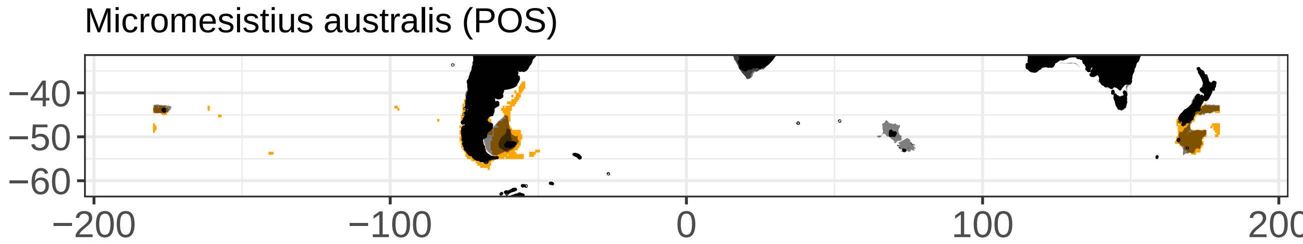
Micromesistius australis (POS)
−40
−50
−60
−200
−100
0
100
200

Micropogonias furnieri (CKM)

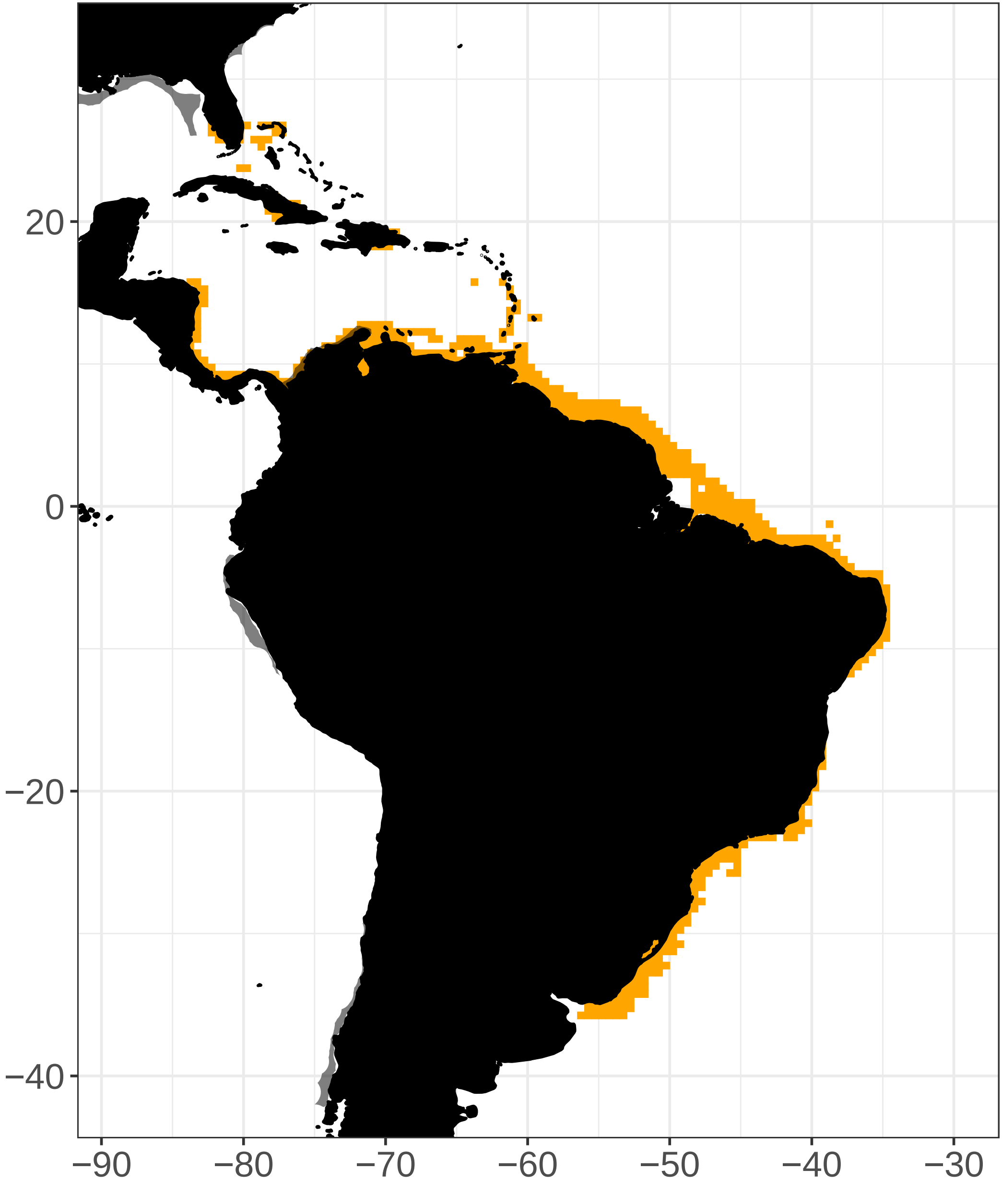

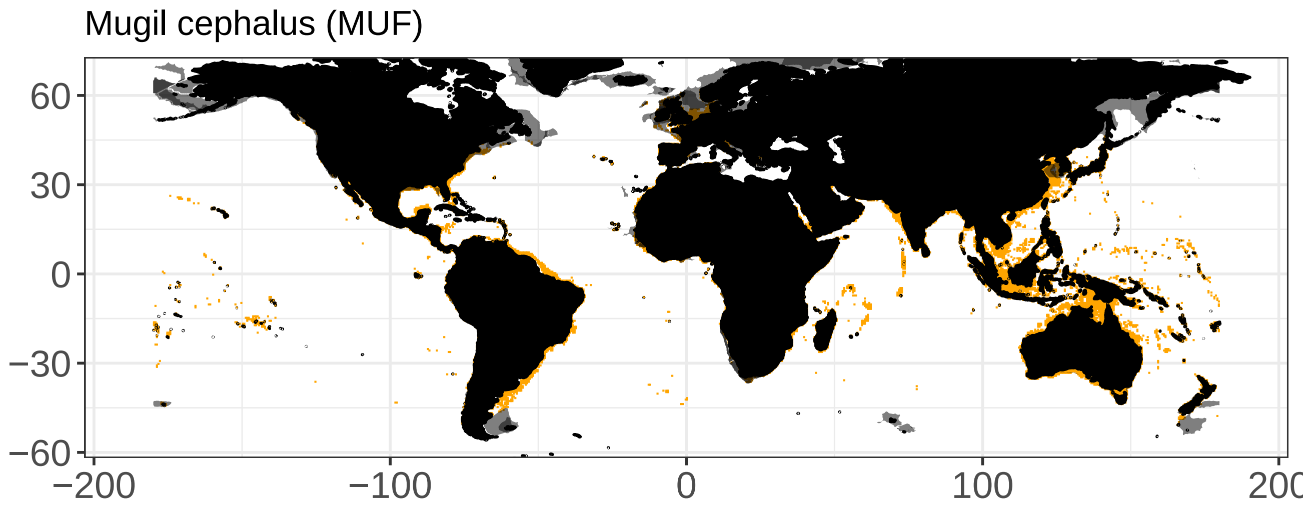
Mugil cephalus (MUF)
60
30
0
−30
−60
−200
−100
0
100
200

Normanichthys crockeri (NRC)

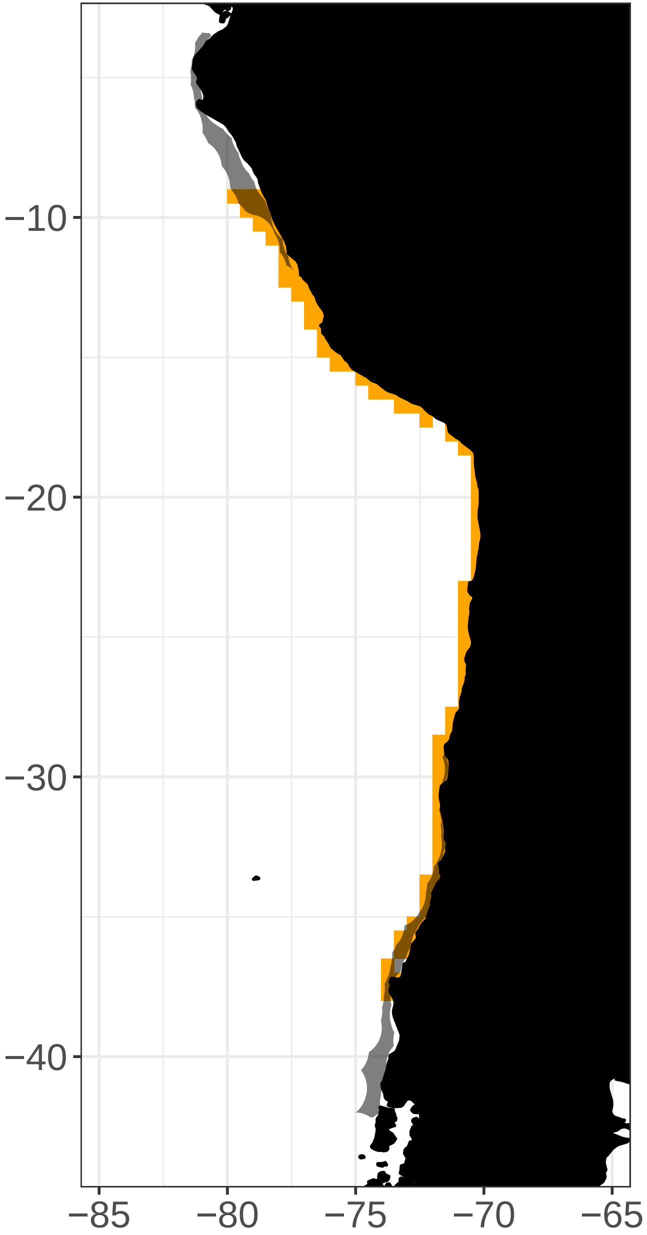

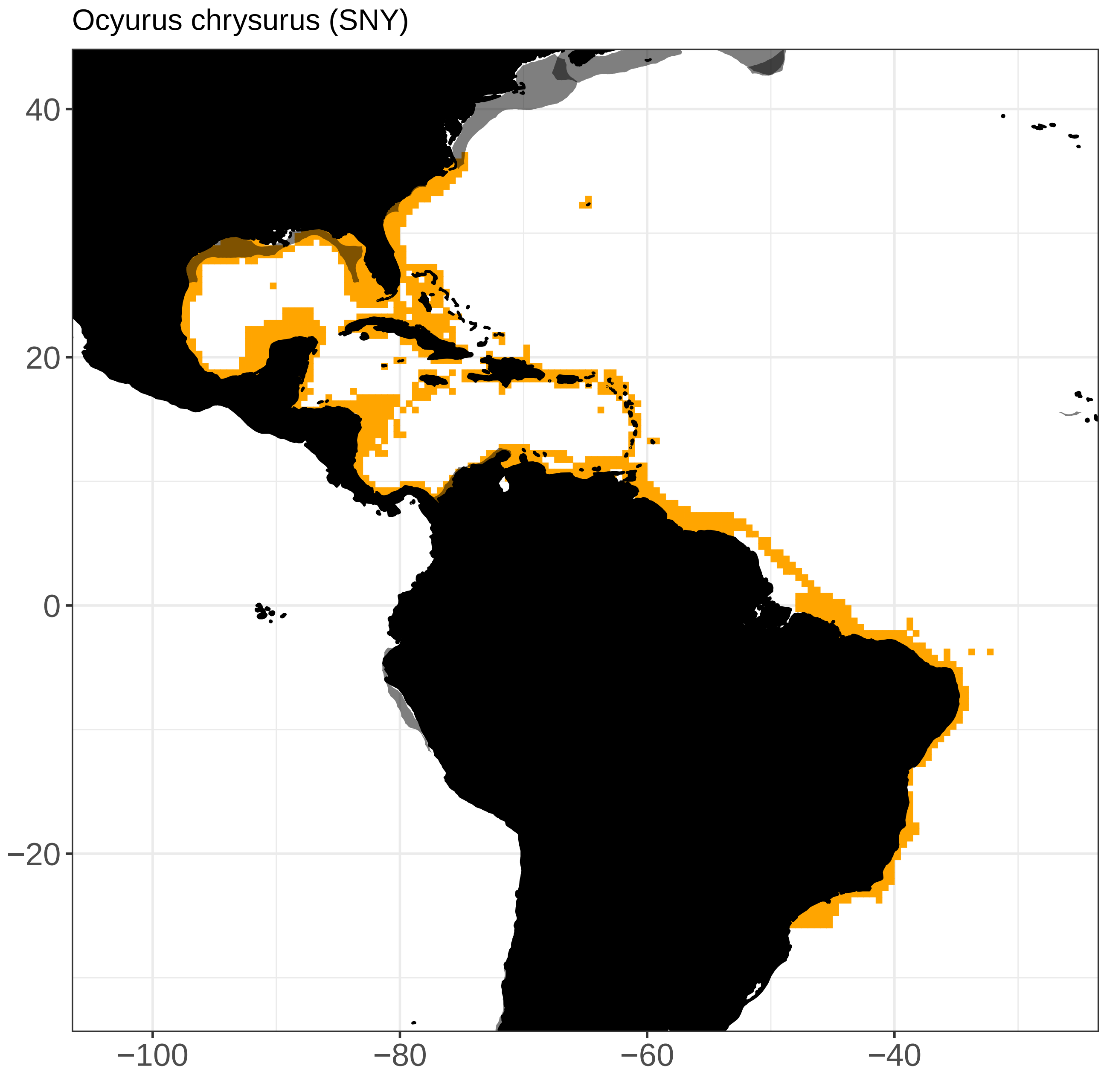
Ocyurus chrysurus (SNY)
40
20
0
−20
−100
−80
−60
−40

Otolithes ruber (LKR)

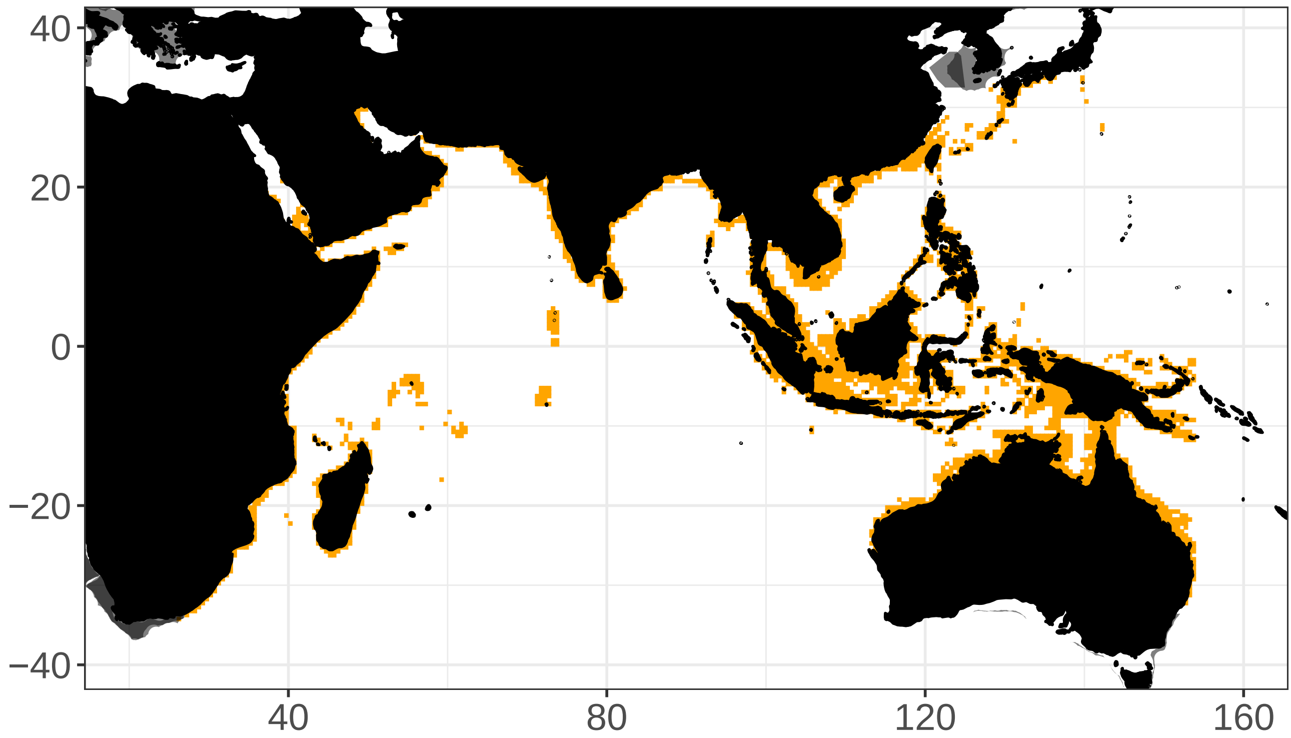

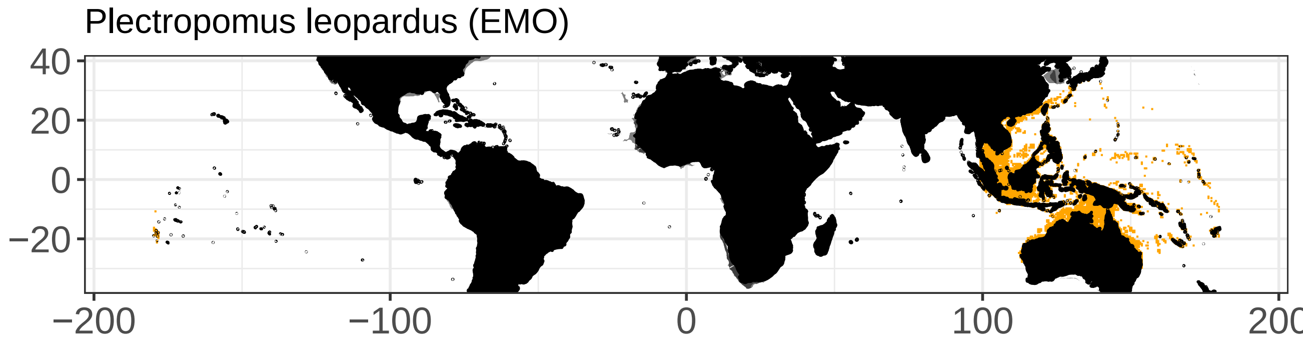
Plectropomus leopardus (EMO)
40
20
0
−20
−200
−100
0
100
200

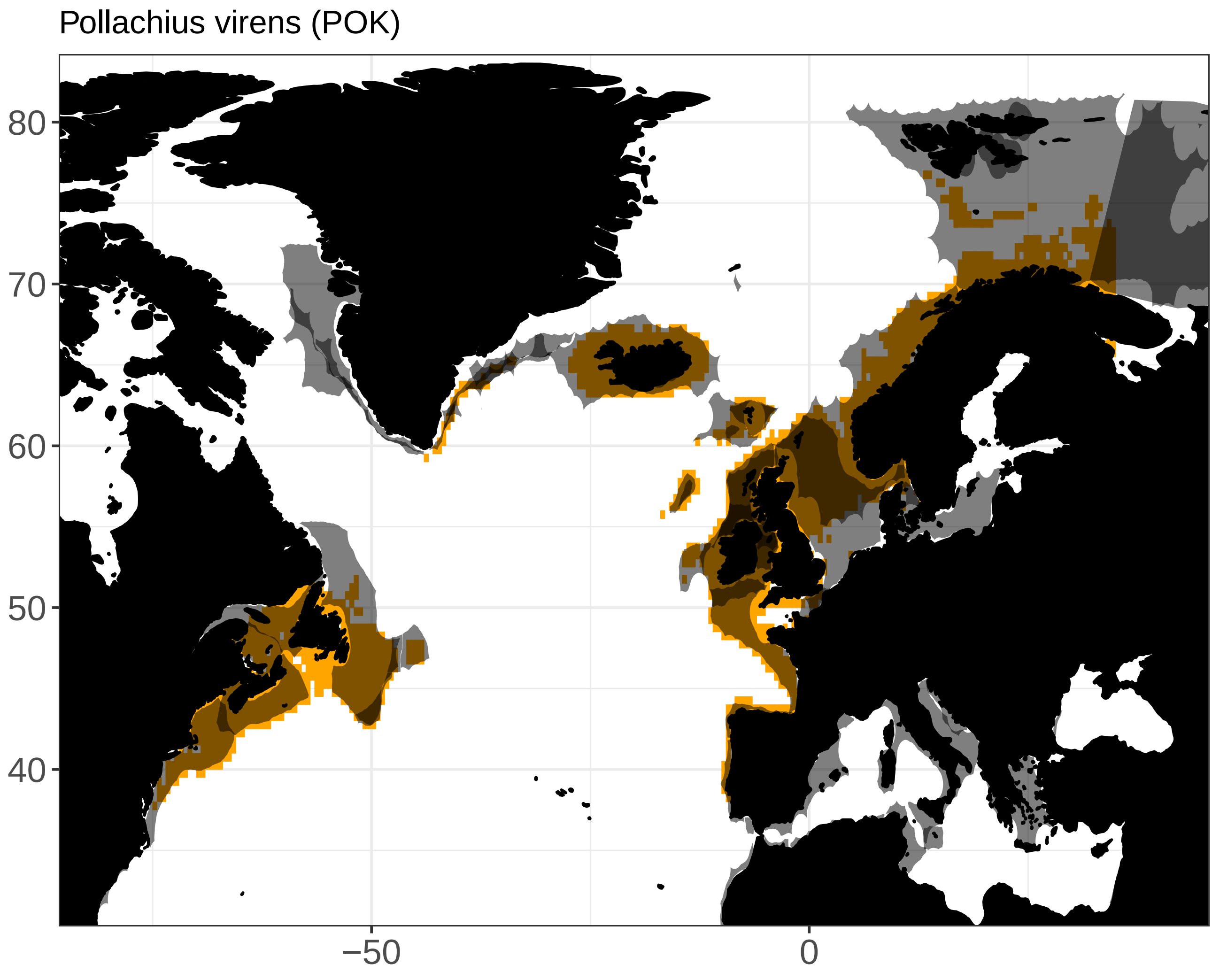
Pollachius virens (POK)
80
70
60
50
40
−50
0

Polydactylus quadrifilis (TGA)

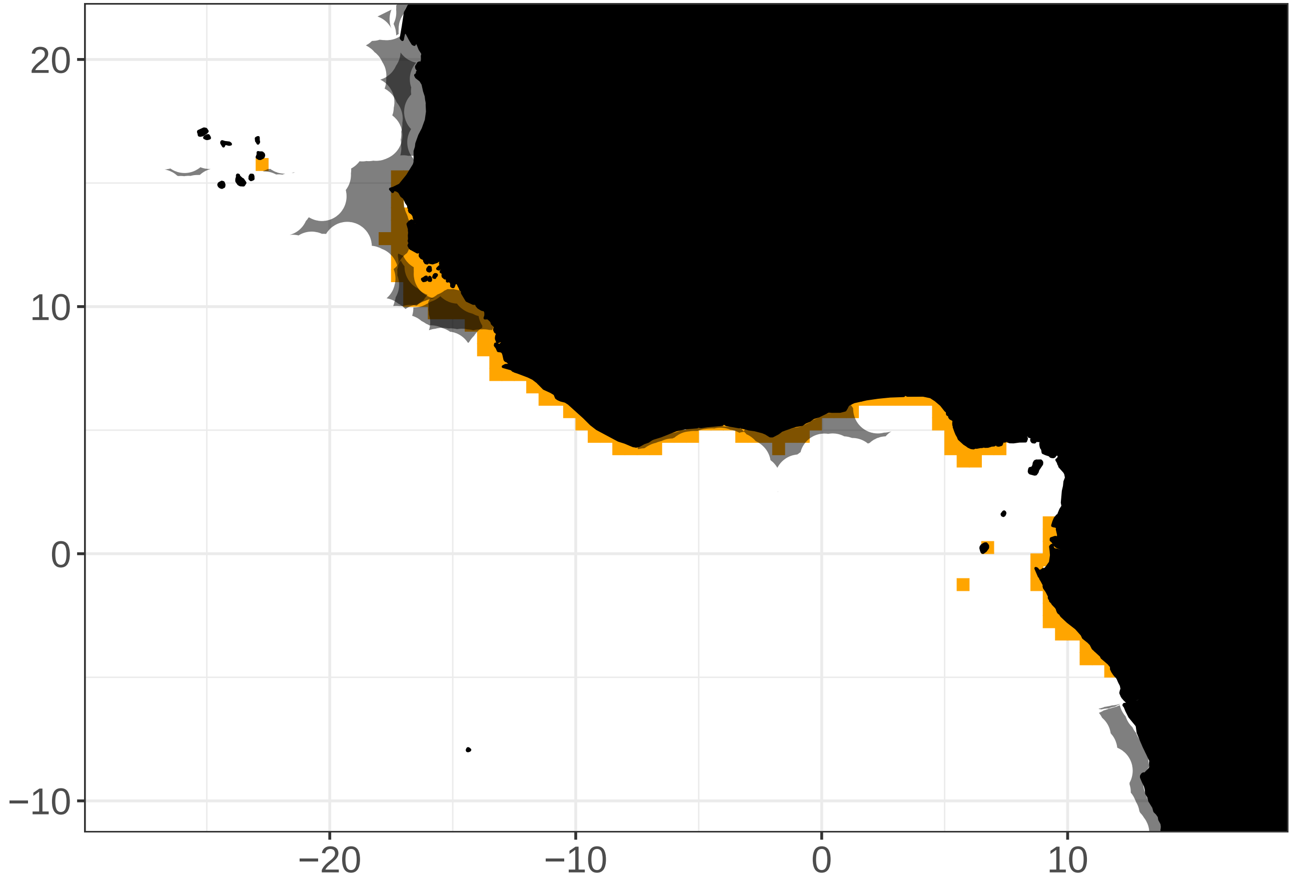

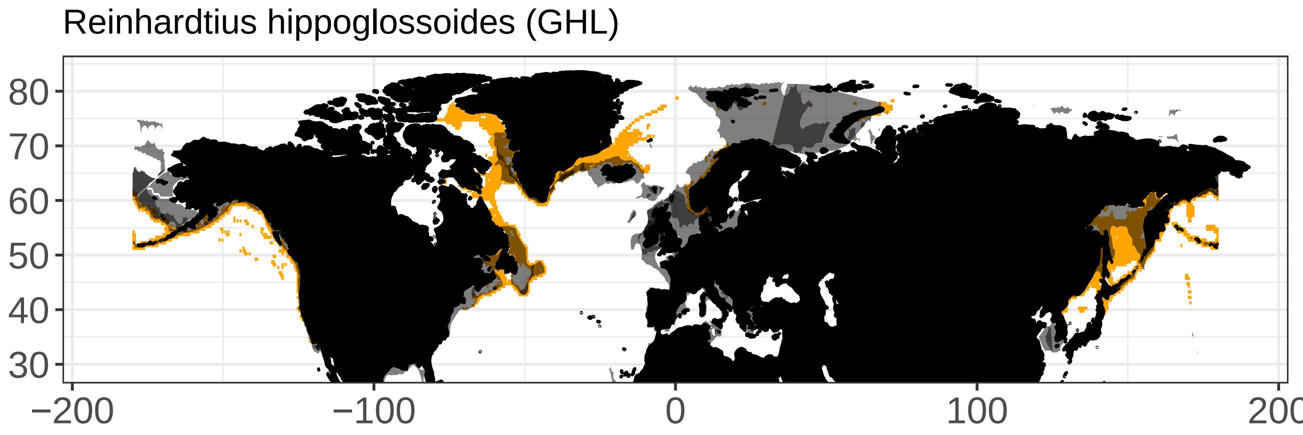
Reinhardtius hippoglossoides (GHL)
80
70
60
50
40
30
−200
−100
0
100
200

Thyrsites atun (SNK)

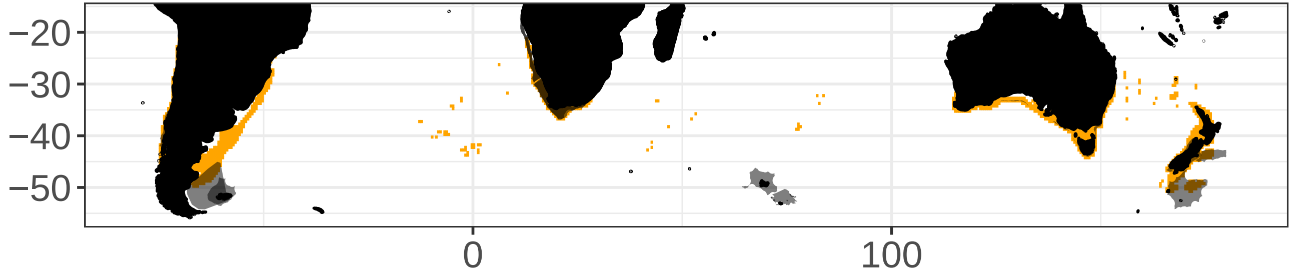

Trichiurus lepturus (LHT)

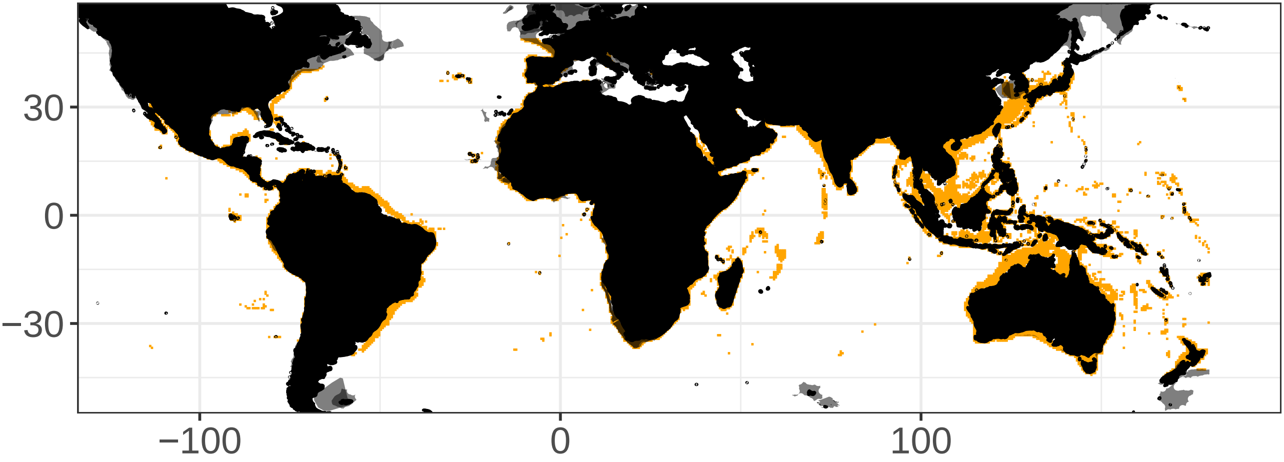

Supplement: Supplementary file 2 — Supplementary Material [file GCB-27-220-s002.pdf]
